# Effect size measure for mediation analysis with a multicategorical predictor

**DOI:** 10.3389/fpsyg.2023.1101440

**Published:** 2023-03-10

**Authors:** Zihuan Cao, Heining Cham, Jordan Stiver, Monica Rivera Mindt

**Affiliations:** Psychology Department, Fordham University, New York, NY, United States

**Keywords:** effect size, mediation analysis, categorical predictor, simulation studies, R-squared

## Abstract

Many currently available effect size measures for mediation have limitations when the predictor is nominal with three or more categories. The mediation effect size measure υ was adopted for this situation. A simulation study was conducted to investigate the performance of its estimators. We manipulated several factors in data generation (number of groups, sample size per group, and effect sizes of paths) and effect size estimation [different *R*-squared (*R*^2^) shrinkage estimators]. Results showed that the Olkin–Pratt extended adjusted *R*^2^ estimator had the least bias and the smallest MSE in estimating υ across conditions. We also applied different estimators of υ in a real data example. Recommendations and guidelines were provided about the use of this estimator.

## Introduction

Mediation analysis has enabled behavioral researchers to better understand the mechanistic relationships between variables. Many researchers are specifically interested in the role of mediators (*M*) that account for the relation between a predictor variable (*X*; independent variable) and an outcome variable (*Y*; dependent variable). A variety of effect size measures have been developed for mediation analysis. However, most of these effect size measures have limitations, including non-monotonicity and spurious inflation. Lachowicz et al. (2018) developed a new effect size measure, upsilon (υ), which has overcome these two limitations. The current study extends its work by applying this effect size metric to mediation models with a multicategorical predictor.

**Figure 1:**
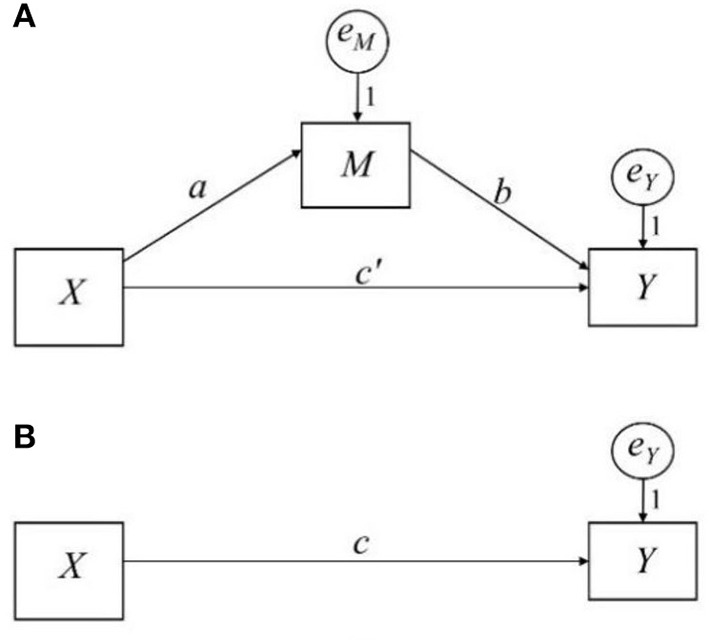
Path diagram of a simple mediation model and the total effect. **(A)** Shows the simple mediation model; **(B)** shows the total effect.

A simple mediation model shown in Figure 1A represents a mediation process in which a predictor *X* indirectly influences an outcome *Y* through a mediator *M*. This causal sequence suggests that *X* exerts a direct effect on *M* (*a* path), which, in turn, causally affects outcome *Y* (*b* path). *X* can have a direct effect on and *Y* (*c*′ path), in which *X* directly influences *Y*.


(1) $$\begin{array}{l} M=i_{1}+aX+e_{M} \end{array}$$

(2) $$\begin{array}{l} Y=i_{2}+c^{\prime }X+bM+e_{Y} \end{array}$$

(3) $$\begin{array}{l} Y=i_{3}+cX+e_{Y} \end{array}$$

(4) $$\begin{array}{l} c=c^{\prime }+ab \end{array}$$

This model can be estimated by a set of regression models or by structural equation modeling when the effects are linear and *M* and *Y* are treated as continuous. Equations 1, 2 are required to estimate the effects of the *a* path and *b* path, respectively. The indirect effect of *X* on *Y* is the product of *a* and *b*. The direct effect is *c*′ (Eq 2). The total effect is *c* in Figure 1B and Eq 3, which equals the sum of *X*'s direct and indirect effects on *Y* (Eq 4).

**Figure 2:**
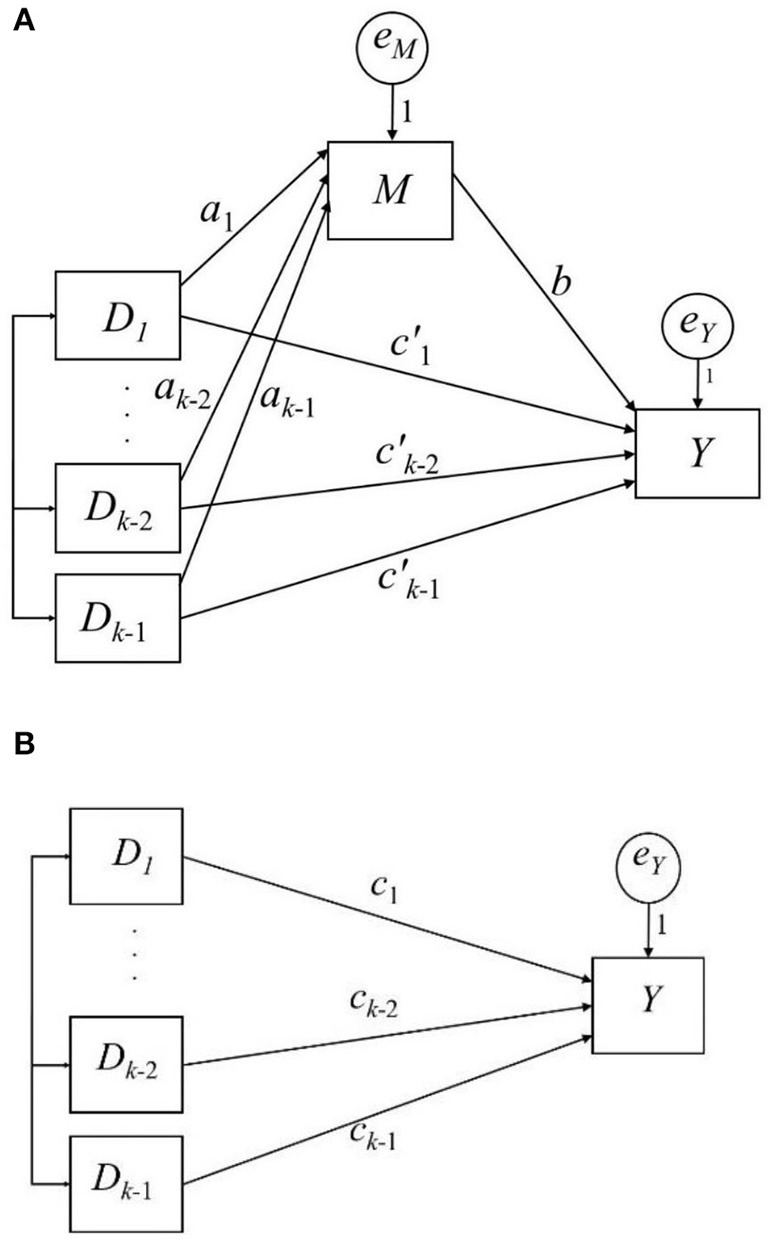
Path diagram of a simple mediation model with a k-Group predictor and the total effect. **(A)** shows the simple mediation model; **(B)** shows the total effect.

In this simple mediation model, predictor *X* can be dichotomous, or it can be treated as continuous. However, mediation analysis with a multicategorical predictor is common (Kalyanaraman and Sundar, 2006). When *X* is multicategorical, it can be expressed by applying coding strategies in regression analysis (Hayes and Preacher, 2014). When there are *k* groups comprising a multicategorical predictor *X*, (*k* − 1) coded variables are computed to represent each group. Different coding strategies are available, and the choice of strategy depends on the research question. Hayes and Preacher (2014) suggested using a dummy or contrast coding for a multicategorical predictor *X* in mediation analysis. In dummy coding, (*k* − 1) dummy variables (*D*_*i*_, *i* = 1, …, *k* − 1) are constructed, where *D*_*i*_ is set to 1 to represent the cases in group *i*; otherwise, it is set to 0. The *k*th group is the reference group and is coded 0 in all *D*_*i*_s (see Table 1 for a three-group example). The effect of *D*_*i*_ is the difference between the *i*th group and the reference group. In contrast coding, *D*_*i*_ is constructed such that its effect represents the difference between the *i*th group and the average of the (*i* + 1)th group to the *k*th group (see Table 1 for a three-group example). A mediation model with a multicategorical predictor *X* is expressed in Figure 2A and Equations 5, 6.


(5) $$\begin{array}{l} M=i_{1}+a_{1}D_{1}+a_{2}D_{2}\ldots +a_{k-1}D_{k-1}+e_{M} \end{array}$$

(6) $$\begin{array}{l} Y=i_{2}+c_{1}^{\prime }D_{1}+c_{2}^{\prime }D_{2}\ldots +c_{k-1}^{\prime }D_{k-1}+bM+e_{Y} \end{array}$$

(7) $$\begin{array}{l} Y=i_{3}+c_{1}D_{1}+c_{2}D_{2}\ldots +c_{k-1}D_{k-1}+e_{Y} \end{array}$$

(8) $$\begin{array}{l} c_{i}=\;c_{i}^{\prime }+a_{i}b,\;where\;i=1\;to\;\left(k\;-\;1\right) \end{array}$$

**Table 1:** Contrast coding for three groups.

**Dummy coding**	**Group 1**	**Group 2**	**Group 3**
D_1_	1	0	0
D_2_	0	1	0
**Contrast coding**	**Group 1**	**Group 2**	**Group 3**
D_1_	2/3	−1/3	−1/3
D_2_	0	1/2	−1/2

Hayes termed *a*_*i*_*b* the relative indirect effect and $c_{i}^{\prime }$ the relative direct effect. Equation 7 and Figure 2B show the relative total effect of *X on Y*, *c*_*i*_, which represents the sum of the corresponding relative direct and indirect effects (Equation 8). The effects are “relative” because they are based on comparisons between the group *i* and the other group (s), depending on the coding strategy of *D*_*i*_.

For statistical inference of the multicategorical mediation analysis, we can use the non-parametric bootstrapping method, Monte Carlo, or product of moments methods to calculate the confidence intervals of relative indirect effects (Hayes and Preacher, 2014). Creedon et al. (2016) developed an omnibus test of the indirect effect of *X* on *Y* through *M*. They suggest using *R*-squared (*R*^2^) to capture the overall effect of *X* on *M* from Equation 5 and construct the confidence interval for the product of this *R*^2^ and *b* to provide an omnibus test of the indirect effect. They tested the performance of this method using Smith, Wherry-1, Wherry-2, Olkin–Pratt, Pratt, and Claudy *R*^2^ shrinkage estimators for *R*^2^ (Creedon et al., 2016), which are supposed to produce less biased estimates for *R*^2^, and the non-parametric bootstrapping method for confidence intervals (*R*^2^ × *b*). While there is no mathematical proof that the product of *R*^2^ and *b* quantifies the overall mediation effect properly, this method has shown satisfactory results in maintaining the type I error rate at a set level (Creedon et al., 2016).

In addition to statistical inferences, reporting on effect sizes is highly encouraged or mandated by many peer-reviewed journals and organizations, including the American American Psychological Association (2010, p. 33) and the International Committee of Medical Journal Editors *via* the Consolidated Standard of Reporting Trials (CONSORT; Moher et al., 2010). Reporting on the effect size of an indirect effect is challenging because mediation analysis is a non-standard regression-based analysis, such that standardized mean differences, correlation coefficients, and proportions of variance explained are effect size metrics that are not sufficient to capture the entirety of an indirect effect (Lachowicz et al., 2018). Preacher (2011) have listed the desiderata for a good effect size measure: (a) An effect size should have an interpretable scale, (b) its confidence interval can be calculated, and (c) its sample estimation of the population parameter should be unbiased, consistent, and efficient. Wen (2015) have since added a desideratum that (d) the function of the effect size measure should be a monotonic representation, either in raw or absolute form, of the quantified effect.

In this study, we focus on a mediation effect size measure υ developed by Lachowicz et al. (2018), which is a modification of MacKinnon's (2008) $R_{4.5}^{2}$ effect size measure. MacKinnon (2008) recommends three proportions of variance-explained measures termed $R_{4.5}^{2}$, $R_{4.6}^{2}$, and $R_{4.7}^{2}$. The subscripts (4.5), (4.6), and (4.7) are based on the original equation numbers in MacKinnon (2008), and these notations have continued to be referenced in subsequent literature (e.g., Lachowicz et al., 2018; Preacher, 2011). In the simple mediation model in Figure 1A, these effect sizes are calculated as follows (Equations 9–11):


(9) $$\begin{array}{l} R_{4.5}^{2}=r_{YM}^{2}-\left(R_{Y,MX}^{2}-r_{YX}^{2}\right) \end{array}$$

(10) $$\begin{array}{l} R_{4.6}^{2}=\left(r_{MX}^{2}\right)\left(r_{YM.X}^{2}\right) \end{array}$$

(11) $$R_{4.7}^{2}=\frac{(r_{MX}^{2})\left(r_{YM.X}^{2}\right)}{R_{Y,MX}^{2}},$$

$where\;r_{YM}^{2}$ is the squared correlation of *Y* and *M*. $r_{YX}^{2}$ is the squared correlation of *Y* and *X*. $R_{Y,MX}^{2}$ is the squared multiple correlations of *Y* jointly explained by *M* and *X*. $r_{MX}^{2}$ is the squared correlation between *M* and *X*. $r_{YM.X}^{2}$ is the squared partial correlation of *Y* and *M* controlling for *X*. $R_{4.5}^{2}$ is the explained variance in *Y* jointly by *M* and *X*. $R_{4.6}^{2}$ is the proportion of variance in *Y* accounted for solely by *M*, weighted by the proportion of explained variance in *M* by *X*. $R_{4.6}^{2}$ is difficult to interpret on an *R*^2^ metric since it is the product of two proportions of variance from different variables (Preacher, 2011). $R_{4.7}^{2}$ is $R_{4.6}^{2}$ divided by $R_{Y,MX}^{2}$, which is interpreted as the proportion of explained variance in *Y* jointly explained by *M* and *X*.

Among the three effect size measures mentioned previously, Lachowicz et al. (2018) modified $R_{4.5}^{2}$ and proposed a new measure υ to address the problem of spurious inflation. To illustrate the issue of spurious inflation, one needs to consider the elements of the simple mediation model in Equations 1–3. It is assumed that *Y* is dependent on *M*, and *Y* and *M* are mutually dependent on *X*, given that all other assumptions hold (temporal precedence, covariation of the cause and effect, etc.,). The zero-order correlation between *M* and *Y* has two components: (a) the conditional correlation between *M* and *Y* independent of *X*, and (b) the correlation due to the mutual dependence of *Y* and *M* on *X*. The first component is often referred to as true correlation, and the latter component is considered spuriously inflated. A true correlation between *M* and *Y* is needed to create a mediation effect size without spurious inflation (Lachowicz et al., 2018). Lachowicz et al. decomposed the correlation between *Y* and *M* (*r*_*YM*_):


(12) $$\begin{array}{l} r_{YM}=\beta_{a}\beta_{c^{\prime }}+\beta_{b}, \end{array}$$

*where β*_*a*_ is the standardized *a* path. $\beta_{c^{\prime }}$ is the standardized *c*′ path. β_*b*_ is the standardized *b* path, which captures the *true* correlation between *M* and *Y* in the mediation model. Lachowicz et al. pointed out that $\beta_{a}\beta_{c^{\prime }}$ is the component that is spuriously inflated, indicating this is a key limitation of $R_{4.5}^{2}$. If there is no indirect effect, either β_*b*_ is zero (i.e., $r_{YM}=\beta_{a}\beta_{c^{\prime }}$), or the inflated term, $\beta_{a}\beta_{c^{\prime }}$, is zero (i.e., β_*a*_ = 0; *r*_*YM*_ = β_*b*_). If there is no direct effect, the spuriously inflated term is zero (i.e., *r*_*YM*_ = β_*b*_). As a result, *r*_*YM*_ cannot distinguish whether an indirect effect is present. Lachowicz et al. (2018) developed the effect size υ by removing the spurious inflation from $R_{4.5}^{2}$; υ measures the variance in *Y* explained jointly by *M* and *X*, correcting for the spurious inflation between *M* and *Y* on *X* (Equation 13). The term ${\left(r_{YM}-\beta_{a}\beta_{c^{\prime }}\right)}^{2}$ is the squared true correlation between *M* and *Y*. $\left(R_{Y,MX}^{2}-r_{YX}^{2}\right)$ is the difference between the total variance in *Y* explained by *M* and *X* ($R_{Y,MX}^{2}$) and the total variance in *Y* explained solely by *X* ($r_{YX}^{2}$). Equation 14 is equal to Equation 13, which replaces ${\left(r_{YM}-\beta_{a}\beta_{c^{\prime }}\right)}^{2}$ with the squared $\beta_{b}^{2}$ from Equation 12. Equation 15 is also equivalent to Equations 13 and 14 (Lachowicz et al., 2018).


(13) $$\upsilon ={\left(r_{YM}-\beta_{a}\beta_{c^{\prime }}\right)}^{2}-\left(R_{Y,MX}^{2}-r_{YX}^{2}\right)$$

(14) $$\begin{array}{l} \upsilon =\beta_{b}^{2}-\left(R_{Y,MX}^{2}-r_{YX}^{2}\right) \end{array}$$

(15) $$\begin{array}{l} \upsilon =\beta_{a}^{2}\;\beta_{b}^{2} \end{array}$$

According to Lachowicz et al., υ has numerous desirable properties as an effect size measure of the indirect effect: (a) It is standardized (scale-free), (b) it is independent of sample size, (c) its function in the absolute value of the indirect effect is monotonic, and (d) its confidence interval can be constructed. Using Equation 15, Lachowicz et al. proposed sample estimators of υ for the simple mediation model (Figure 1A). The first estimator is $\hat{\upsilon }={\hat{\beta }}_{a}^{2}{\hat{\beta }}_{b}^{2}$ where $\hat{•}$ is the sample estimator. Their simulation study found that it was upwardly biased. Based on the relationship that $E\left(\hat{B^{2}}\right)=B^{2}+\sigma_{B}^{2}$, where *E*(•) is the expectation function, *B* is the unstandardized regression coefficient, and $\sigma_{B}^{2}$ is the variance of $\hat{B}$, they propose the second estimator:


(16) $$\begin{array}{l} \hat{\upsilon }=\left({â}^{2}-\sigma_{a}^{2}\right)\left({\hat{b}}^{2}-\sigma_{b}^{2}\right)\left(\frac{{\hat{\sigma }}_{X}^{2}}{{\hat{\sigma }}_{Y}^{2}}\right). \end{array}$$

where $\sigma_{a}^{2}$ and $\sigma_{b}^{2}$ are the variances of â and $\hat{b}$, respectively. ${\hat{\sigma }}_{X}^{2}$and ${\hat{\sigma }}_{Y}^{2}$ are the sample variances of *X* and *Y*. The confidence interval of υ can be calculated *via* non-parametric bootstrapping. Their simulation study found that this estimator had an acceptable bias with only four experimental conditions resulting in percent relative biases > 5% at *N* = 100. The confidence interval of υ can be constructed using this estimator *via* non-parametric bootstrapping; their simulation showed that the bootstrapped confidence interval had an acceptable coverage rate (i.e., between 92.5 and 97.5%) at *N* = 250.

## Current study

Because of the desirable properties of υ, we applied it in a simple mediation model with a multicategorical predictor (Figure 2A). We conducted a simulation study to investigate the performance of a sample estimator of υ. Using Equation 15, the υ for each relative indirect effect *a*_*i*_*b* equals $\beta_{a_{i}}^{2}\;\beta_{b}^{2}$, where β_*a*_*i*__ is the standardized *a*_*i*_ path and β_*a*_*i*__ = *a*_*i*_(σ_*D*_*i*__/σ_*M*_). In addition, we believe that researchers would be interested in calculating υ for the overall indirect effect of *X* on *Y* through *M*. Equation 15 and its sample estimators are difficult to apply in this situation because of multiple *a*_*i*_ paths; thus, we chose Equation 14 and proposed an estimator of υ:


(17) $$\begin{array}{l} \tilde{\upsilon }=\left({\hat{b}}^{2}-\sigma_{b}^{2}\right)\left(\frac{{\hat{\sigma }}_{M}^{2}}{{\hat{\sigma }}_{Y}^{2}}\right)-\left({\tilde{R}}_{Y,MX}^{2}-{\tilde{r}}_{YX}^{2}\right). \end{array}$$

Following Equation 16, we chose $\left({\hat{b}}^{2}-\sigma_{b}^{2}\right)\left(\frac{{\hat{\sigma }}_{M}^{2}}{{\hat{\sigma }}_{Y}^{2}}\right)$ in the hope of getting a less biased estimate of $\beta_{b}^{2}$. ${\tilde{R}}_{Y,MX}^{2}$ and ${\tilde{r}}_{YX}^{2}$ are the shrinkage estimators of $R_{Y,MX}^{2}$ and $r_{YX}^{2}$, respectively. Following Creedon et al. (2016), we hoped that the shrinkage estimators would provide less biased estimates of $R_{Y,MX}^{2}$ and $r_{YX}^{2}$. Therefore, all the components in Equation 14 (i.e., population υ) were adjusted for small-sample biases in Equation 17.

Table 2 summarizes the formulas of the shrinkage estimators to adjust *R*^2^ and *r*^2^ (i.e., *R*^2^ in single-predictor regression). Based on the results from previous simulation studies on the performance of the shrinkage estimators (Raju et al., 1999; Yin, 2001; Walker, 2007; Wang and Thompson, 2007; Shieh, 2008; Creedon et al., 2016), Pratt, Ezekiel, Smith, Wherry-2, Walker, and Olkin–Pratt extended formulas performed well in at least one study. Nevertheless, none of these studies directly tested the mediation effect size measures. It is difficult to determine which formula is the best option for the sample adjustments of $R_{Y,MX}^{2}$ and $r_{YX}^{2}$ when estimating υ. We conducted a simulation study to examine the following shrinkage estimators: Claudy, Ezekiel, Olkin–Pratt, Olkin–Pratt extended, Pratt, Smith, Walker, and Wherry.

**Table 2:** Formulas of *R*^2^ shrinkage estimators.

**Shrinkage estimator**	**Formula**
Claudy (Claudy-3)	${\hat{\rho }}_{\left(C\right)}^{2}=1-\frac{\left(N-4\right)\left(1-R^{2}\right)}{N-p-1}\;\left[1+\frac{2\left(1-R^{2}\right)}{N-p+1}\right]$ ${\hat{\rho }}_{\left(E\right)}^{2}=1-\frac{N-1}{N-p-1}\left(1-R^{2}\right)$ ${\hat{\rho }}_{\left(C\right)}^{2}=1-\frac{\left(N-3\right)\left(1-R^{2}\right)}{N-p-1}\;\left[1+\frac{2\left(1-R^{2}\right)}{N-p+1}\right]$ ${\hat{\rho }}_{\left(OP\right)}^{2}=1-\frac{\left(N-3\right)\left(1-R^{2}\right)}{N-p-1}\;\left[1+\frac{2\left(1-R^{2}\right)}{N-p+1}\right]$ $r_{OP}^{2}={\left(r\times \;\left[1+\frac{\;1-r^{2}\;}{2\left(N-3\right)}\right]\;\right)}^{2}$ ${\hat{\rho }}_{\left(OPE\right)}^{2}=1-\frac{\left(N-3\right)\left(1-R^{2}\right)}{N-p-1}\;\left[1+\frac{2\left(1-R^{2}\right)}{N-p+1}+\frac{8{\left(1-R^{2}\right)}^{2}}{\left(N-p-1\right)\left(N-p+3\right)}\right]$ ${\hat{\rho }}_{\left(P\right)}^{2}=1-\frac{\left(N-3\right)\left(1-R^{2}\right)}{N-p-1}\;\left[1+\frac{2\left(1-R^{2}\right)}{N-p-2.3}\right]$ ${\hat{\rho }}_{\left(S\right)}^{2}=1-\frac{N}{N-p}\left(1-R^{2}\right)$ ${\hat{\rho }}_{\left(DW\right)}^{2}=1-\frac{\left(N-4.15\right)\left(1-R^{2}\right)}{N-p-1}\;-\frac{2\left(N-4.15\right)\left(1-R^{2}\right)}{\left(N-p-1\right)\left(N-p+1\right)}$ ${\hat{\rho }}_{\left(W\right)}^{2}=1-\frac{N-1}{N-p}\left(1-R^{2}\right)$
Ezekiel (Wherry-1)	
Herzberg	
Olkin-Pratt	
Olkin-Pratt Pearson *r*^2^	
Olkin-Pratt Extended	
Pratt	
Smith	
Walker	
Wherry (Wherry-2)	

*N* is sample size. *p* is number of predictors. *R*^2^ is the sample squared multiple correlation. *r* is the sample correlation.

## Methods

A simulation study was conducted to evaluate the performance of sample estimator υ in Equation 17 under finite samples. The simulation was based on the mediation model in Figure 2A. Five factors were manipulated:

(1) A number of groups in *X*: *k* = 3, 4, 5. The conditions followed those in Creedon et al. (2016).
(2) Sample size per group, *n* = 10, 20, 25, 50, 100. The range of *n* covered small-to-large sample sizes across groups.
(3) Effect size of *a*_*i*_ paths were manipulated as the mean difference between adjacent groups on *M*. The mean difference was set in terms of Cohen's *d* = 0, 0.2, 0.5, 0.8, representing null, small, medium, and large effects, respectively (Cohen, 1992).
(4) Size of *b* path, *b* = 0, 0.15, 0.39, 0.59. The conditions followed those in Lachowicz et al. (2018).
(5) Effect size of $c_{i}^{\prime }$ paths were manipulated as the mean difference between adjacent groups on *Y*. The mean difference was set in terms of Cohen's *d* = 0.1, 0.2, 0.3.

In all conditions, the residual of the mediator *M*, *e*_*M*_, was a normal variable with variance determined by $R_{MX}^{2}$, and the residual of the outcome *Y*, *e*_*Y*_, was a standard normal variable. The simulation used a full factorial design with a total of 720 conditions (3 × 5 × 4 × 4 × 3). For each condition, 10,000 replications were created. For each condition and replication, nine $R_{Y,MX}^{2}$ and $r_{YX}^{2}$ estimators were used to estimate υ: unadjusted, Claudy, Ezekiel, Olkin–Pratt, Olkin–Pratt extended, Pratt, Smith, Walker, and Wherry. The unadjusted sample estimates of υ were calculated using Equation 14, in which β_*b*_, $R_{Y,MX}^{2}$, and $r_{YX}^{2}$ were not adjusted. The rest of the estimators were calculated using Equation 17. The 95% confidence interval of υ was constructed using non-parametric bootstrapping with 1,000 bootstrap samples. The simulation study was conducted in *R* (Version 3.5.3; Windows system), and the packages *boot 1.3–20* (Canty, 2017), *dummies 1.5.6* (Brown, 2012), *effsize 0.7.4* (Torchiano, 2018), and *lm.beta 1.5–1* (Behrendt, 2014) were utilized.

To evaluate the performance of sample estimators of υ, the following outcomes were used: bias, standardized bias, mean squared error (MSE), and coverage rate. For any parameter θ, bias is the difference between the expectation of the sample estimates $\hat{\theta }$ and the parameter (Equation 18).


(18) $$\begin{array}{l} bias\left(\hat{\theta }\right)=E\left(\hat{\theta }\right)-\theta . \end{array}$$

In each condition, the population value of υ was calculated using Equation 14. The online Supplementary material present the population value of υ in each simulation condition.

Standardized bias is the bias divided by the standard deviation of the sample estimates $\hat{\theta }$ (Equation 19). Standardized bias within ±0.4 can be regarded as acceptable (Collins et al., 2001).


(19) $$\begin{array}{l} \text{standardized bias}\left(\hat{\theta }\right)=\frac{E\left(\hat{\theta }\right)-\;\theta }{SD\left(\hat{\theta }\right)}. \end{array}$$

Mean squared error is the expected squared difference between the sample estimates and the parameter. It is equal to the sum of the variance of sample estimates and squared bias (Equation 20). An unbiased sample estimator would produce an MSE equal to the variance of the estimator.


(20) $$\begin{array}{l} {MSE=E{\left(\hat{\theta }-\theta \right)}^{2}=\sigma_{\theta }^{2}+\left[bias\left(\hat{\theta }\right)\right]}^{2}. \end{array}$$

Coverage rate is the proportion of the sample in which the population parameter is contained within the 95% confidence interval across replications in a condition. An acceptable coverage rate is between 92.5 and 97.5% (Bradley, 1978).

We expected that the unadjusted $\hat{\upsilon }$ would produce upwardly biased estimates, particularly for small sample size (*N*) and small effect size conditions. The bias of unadjusted $\hat{\upsilon }$ would decrease with increasing *N* and effect size. For the shrinkage adjusted $\tilde{\upsilon }$, it was expected that the estimates would have acceptable bias and that the changes in *N* would not change the bias. We expected that the MSE of the unadjusted $\hat{\upsilon }$ would decrease as *N* increased since larger sample sizes decrease both sampling error and bias. For the same reason, we expected that the MSE of the shrinkage adjusted $\tilde{\upsilon }$ would also decrease as *N* increased. Finally, for both unadjusted $\hat{\upsilon }$ and shrinkage adjusted $\tilde{\upsilon }$, we expected that the coverage rate would approach the acceptable range as *N* increased.

We conducted one-way analyses of variance (ANOVAs) to study the effects of each manipulated factor (number of groups, sample size per group, effect size of *a*_i_ paths, size of *b* path, and effect size of $c_{i}^{\prime }$ paths) on the bias, standardized bias, MSE, and coverage rate. For each significant factor, we conducted *post-hoc* pairwise comparisons with Tukey's HSD tests and produced boxplots by different conditions within the factor.

## Results

Table 3 shows the bias, standardized bias, MSE, and coverage rate of $\tilde{\upsilon }\;$using different *R*^2^ shrinkage method across a number of groups, group sizes, and sizes of *a*_*i*_, *b*, and $c_{i}^{\prime }$ paths. ANOVAs and *post hoc* pairwise comparison results of each $\tilde{\upsilon }$ estimator are provided in the online supplements. All the *R*^2^ shrinkage methods performed similarly. The Olkin–Pratt extended method had the least bias and the lowest MSE. The Smith method yielded the least standardized bias. However, the unadjusted method and all the *R*^2^ shrinkage methods produced very large standardized biases and were beyond the acceptable range (>0.4). None of the estimators produced satisfactory coverage rates (>0.9) across conditions. The unadjusted $\hat{\upsilon }$ had the highest coverage rate among all the sample estimators. This finding was consistent with Lachowicz et al. (2018). Based on the results, we decided to focus on the Olkin–Pratt extended shrinkage method (${\tilde{\upsilon }}_{OPE}$) hereafter. The online supplements provide the results of ANOVAs and *post hoc* pairwise comparisons of each manipulated factor on the bias, standardized bias, MSE, and coverage rate of ${\tilde{\upsilon }}_{OPE}$.

**Table 3:** Comparison of sample estimators of υ across all conditions.

**Sample estimator**	**Bias**	**Standardized relative bias**	**MSE**	**Coverage rate (%)**
Unadjusted $\hat{\upsilon }$	0.02314	0.56756	0.00430	71.67
Claudy adjusted $\tilde{\upsilon }$ (${\tilde{\upsilon }}_{Claudy}$)	0.02191	0.64609	0.00445	66.62
Ezekiel adjusted $\tilde{\upsilon }$ (${\tilde{\upsilon }}_{Ezekiel\;}$)	0.02184	0.62681	0.00439	63.48
Olkin-Pratt adjusted $\tilde{\upsilon }$ (${\tilde{\upsilon }}_{OP}$)	0.02103	0.65311	0.00426	67.43
Olkin-Pratt Extended adjusted $\tilde{\upsilon }$ (${\tilde{\upsilon }}_{OPE}$)	0.02080	0.65121	0.00422	67.64
Pratt adjusted $\tilde{\upsilon }$ (${\tilde{\upsilon }}_{Pratt\;}$)	0.02088	0.66614	0.00423	67.54
Smith adjusted $\tilde{\upsilon }$ (${\tilde{\upsilon }}_{Smith\;}$)	0.02171	0.52229	0.00439	66.79
Walker adjusted $\tilde{\upsilon }$ (${\tilde{\upsilon }}_{Walker\;}$)	0.02204	0.64510	0.00448	66.44
Wherry adjusted $\tilde{\upsilon }$ (${\tilde{\upsilon }}_{Wherry}$)	0.02253	0.52666	0.00457	64.84

### Bias

**Figure 3:**
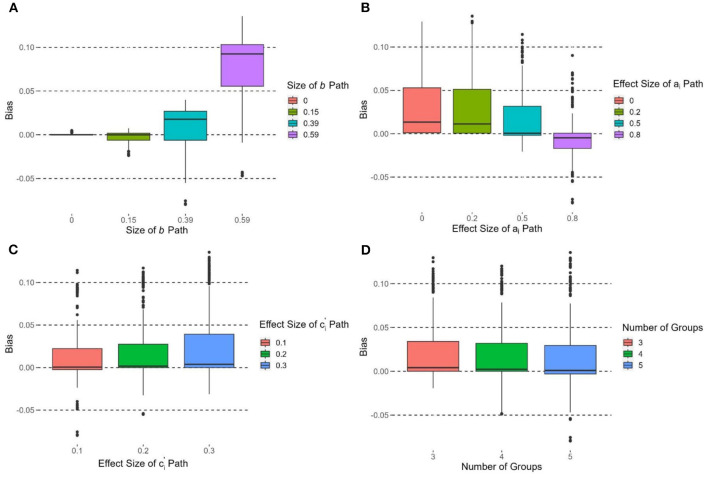
Boxplots of bias of ${\tilde{\upsilon }}_{OPE}$ grouped by **(A)** size of *b* path, **(B)** effect size of a_i_ path, **(C)** effect size of ${\text{c}}_{\text{i}}^{\prime }$ path, and **(D)** number of groups.

The size of the *b* path had the strongest effect on the bias of ${\tilde{\upsilon }}_{OPE}$, *F*(3, 716) = 448.22, *p* < 0.0001; η^2^ = 0.65. Figure 3A shows the boxplots of bias of ${\tilde{\upsilon }}_{OPE}$ by different *b* path conditions. The bias of ${\tilde{\upsilon }}_{OPE}$ at *b* = 0.59 was significantly higher than all the other *b* path conditions. The bias at *b* = 0.39 was also significantly higher than the *b* = 0.15 and *b* = 0 conditions. A higher effect of *b* path was associated with a more positive bias ${\tilde{\upsilon }}_{OPE}$.

The effect size of *a*_*i*_ paths had the second strongest effect on the bias of ${\tilde{\upsilon }}_{OPE}$, *F*(3, 716) = 39.13, *p* < 0.0001, η^2^ = 0.14. Figure 3B shows the boxplots of bias of ${\tilde{\upsilon }}_{OPE}$ by different effect sizes of *a*_*i*_ paths. All pairwise comparisons were significant (*p*s < 0.05) except (*d* = 0.2 vs. *d* = 0). The bias of ${\tilde{\upsilon }}_{OPE}$ at *d* = 0.8 was significantly lower than *d* = 0.5, 0.2, and 0. Bias of ${\tilde{\upsilon }}_{OPE}$ was the lowest when *d* = 0.5. The effect size of $c_{i}^{\prime }$ paths were also affected by the bias of ${\tilde{\upsilon }}_{OPE}$ with *F*(2, 717) = 8.26, *p* < 0.001 η^2^ = 0.02. Figure 3C shows that the larger effect sizes of $c_{i}^{\prime }$ paths had a larger bias of ${\tilde{\upsilon }}_{OPE}$. The number of groups had a small effect on the bias of ${\tilde{\upsilon }}_{OPE}$, *F*(2, 717) = 3.20, *p* < 0.05, η^2^ = 0.009 (Figure 3D). The bias of ${\tilde{\upsilon }}_{OPE}$ at groups of *k* = 5 was significantly smaller than that at *k* = 3, and the bias was the lowest at *k* = 5. Group size had no significant effect on the bias of ${\tilde{\upsilon }}_{OPE}$, *F*(4, 715) = 0.57, *p* = 0.69, η^2^ = 0.003.

### Standardized bias

**Figure 4:**
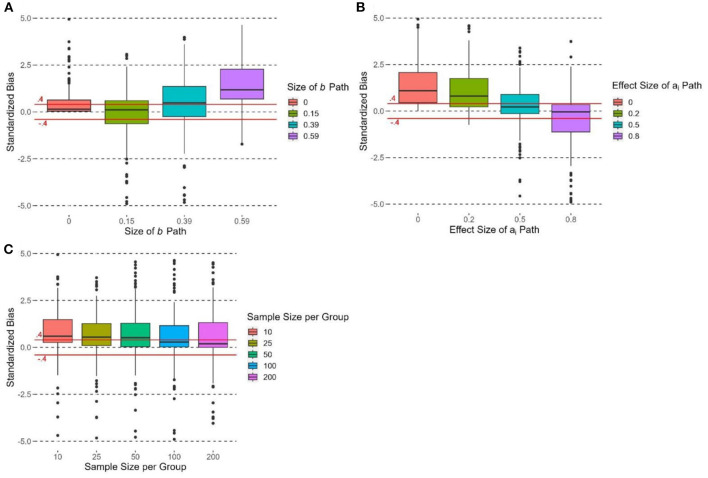
Boxplots of standardized bias of ${\tilde{\upsilon }}_{OPE}$ grouped by **(A)** size of *b* path, **(B)** effect size of a_i_ path, and **(C)** sample size per group. Due to the limit of y-axis, 6.53% of the observations are not shown in each box plot; the acceptable range of standardized bias (i.e., between −0.4 and 0.4) is indicated by the solid horizontal lines.

The size of the *b* path had an effect on the standardized bias of ${\tilde{\upsilon }}_{OPE}$, *F*(3, 716) = 25.73, *p* < 0.0001, η^2^ = 0.10. ${\tilde{\upsilon }}_{OPE}$ had the most positive standardized bias when *b* = 0.59 with (Figure 4A). When *b* = 0.39, ${\tilde{\upsilon }}_{OPE}$ had more than 50% of cases with positive standardized bias > 0.4. When *b* = 0.15 and 0, the median standardized bias was within the ±0.4 criterion, yet some cases were beyond this range. The effect size of *a*_*i*_ paths had an effect on the standardized bias of ${\tilde{\upsilon }}_{OPE}$, *F*(3, 716) = 19.98, *p* < 0.0001, η^2^ = 0.08. Figure 4B shows that the standardized bias of ${\tilde{\upsilon }}_{OPE}$ decreased when the effect size of *a*_*i*_ paths increased. When *d* = 0 and 0.2, the standardized bias of ${\tilde{\upsilon }}_{OPE}$ was > 0.4. When *d* = 0.5, the median standardized bias was < 0.4. When *d* = 0.8, the standardized bias of ${\tilde{\upsilon }}_{OPE}$ was the lowest and the median standardized bias was close to 0. The effect size of $c_{i}^{\prime }$ paths did not have a significant effect on the standardized bias of ${\tilde{\upsilon }}_{OPE}$, *F*(2, 717) = 0.25, *p* = 0.78, η^2^ = 0.001.

The number of groups had no significant effect on the standardized bias of ${\tilde{\upsilon }}_{OPE}$, *F*(2, 717) = 2.84, *p* = 0.06, η^2^ = 0.008. Group size had a significant effect on the standardized bias of ${\tilde{\upsilon }}_{OPE}$, *F*(4, 715) = 7.66, *p* < 0.0001, η^2^ = 0.04. Figure 4C shows that the standardized bias decreased when group size increased. The standardized bias of ${\tilde{\upsilon }}_{OPE}$ was the most positive when *n*= 10 (median > 0.4), and its standardized bias was significantly more positive than those in other conditions (*p*s < 0.05). The median standardized bias was >0.4 when group size = 25 and 100. When group size = 100 and 200, the median standardized bias was < 0.4.

### Mean squared error

**Figure 5:**
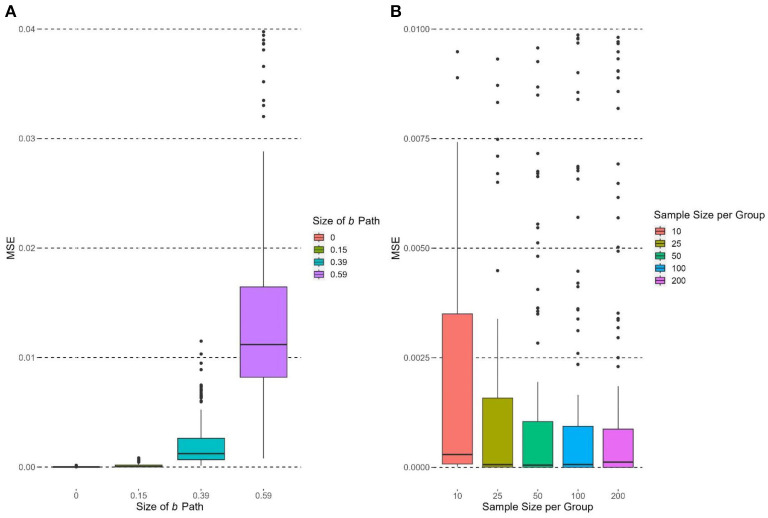
Boxplots of MSE of ${\tilde{\upsilon }}_{OPE}$ grouped by experimental conditions. Due to limit of y-axis, 0.97% and 15.83% of the observations are not shown in **(A)** and **(B)**, respectively.

The size of the *b* path had a significant and large effect on the MSE of ${\tilde{\upsilon }}_{OPE}$, *F*(3, 716) = 301.85, *p* < 0.0001, η^2^ = 0.56. MSE of ${\tilde{\upsilon }}_{OPE}$ increased when *b* increased (Figure 5A). The MSE at *b* = 0.59 was significantly higher than those in other conditions (*b* = 0.39, 0.15, 0; *p*s < 0.05), and the MSE at *b* = 0.39 was significantly higher than those at *b* = 0.15 and 0 (*p*s < 0.05). When *b* = 0 and 0.15, the MSE were close to zero. The effect sizes of *a*_*i*_ and $c_{i}^{\prime }$ paths did not have significant effects on the MSE of ${\tilde{\upsilon }}_{OPE}$ [*a*_*i*_: *F*(3, 716) = 0.90, *p* = 0.44, η^2^ = 0.004; $c_{i}^{\prime }$: *F*(2, 717) = 2.29, *p* = 0.10, η^2^ = 0.006].

The number of groups had no significant effect on the MSE of ${\tilde{\upsilon }}_{OPE}$, *F*(2, 717) = 1.11, *p* = 0.33, η^2^ = 0.003. Group size had a significant effect on the MSE of ${\tilde{\upsilon }}_{OPE}$, *F*(4, 715) = 23.18, *p* < 0.0001, η^2^ = 0.12. Figure 5B supports the hypothesis that the MSE of ${\tilde{\upsilon }}_{OPE}$ would decrease as group size increased in general. MSE of ${\tilde{\upsilon }}_{OPE}$ was the highest when group size = 10, and its MSE was significantly higher than those in other conditions (*p*s < 0.05). When group size = 25, 50, and 100, the median MSEs were close to zero.

### Coverage rate

**Figure 6:**
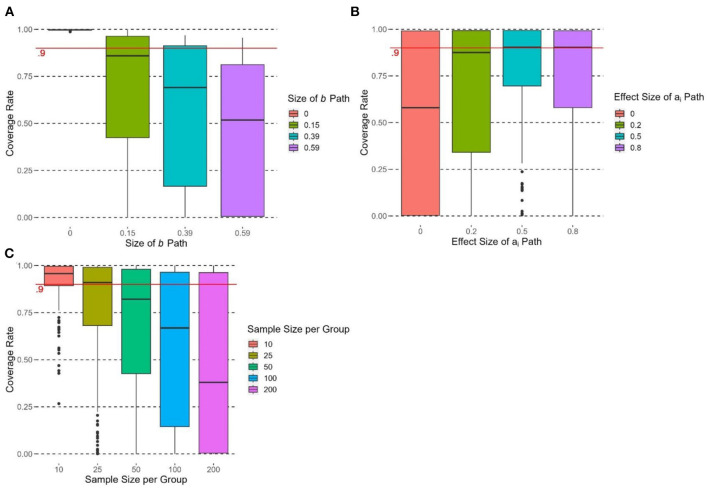
Boxplots of coverage rate of ${\tilde{\upsilon }}_{OPE}$ grouped by **(A)** size of *b* path, **(B)** effect size of a_i_ path, and **(C)** sample size per group. The cutoff of satisfactory coverage rate (coverage rate > 0.9) is indicated by the solid horizontal line.

The size of the *b* path had a significant effect on the coverage rate of ${\tilde{\upsilon }}_{OPE}$, *F*(3, 716) = 99.18, *p* < 0.0001, η^2^ = 0.29. All pairwise comparisons were significant (*p*s < 0.05). Figure 6A shows that the coverage rate decreased when *b* increased, and only when *b* = 0 did ${\tilde{\upsilon }}_{OPE}$ reach satisfactory coverage rate (>0.9). The effect size of *a*_*i*_ paths had a significant effect on the coverage rate of ${\tilde{\upsilon }}_{OPE}$, *F*(3, 716) = 2.24, *p* < 0.0001, η^2^ = 0.08. Figure 6B shows that coverage rate at *d* = 0 had a significantly lower coverage rate than all other conditions (*d* = 0.2, 0.5, 0.8; *p*s < 0.05). For *d* = 0.2, 0.5, and 0.8 conditions, ${\tilde{\upsilon }}_{OPE}$ did not have satisfactory coverage rates (< 0.9). The effect size of $c_{i}^{\prime }$ paths did not have a significant effect on the coverage rate of ${\tilde{\upsilon }}_{OPE}$, *F*(2, 717) = 0.01, *p* = 0.99, η^2^ < 0.0001. None of the conditions for $c_{i}^{\prime }$ paths reached a majority of satisfactory coverage rates.

The number of groups had no significant effect on the coverage rate of ${\tilde{\upsilon }}_{OPE}$, *F*(2, 717) = 1.83, *p* = 0.16, η^2^ = 0.005. Group size had a significant effect on the coverage rate of ${\tilde{\upsilon }}_{OPE}$, *F*(4, 715) = 36.26, *p* < 0.0001, η^2^ = 0.17. Figure 6C shows that the coverage rate decreased when the group size increased. The coverage rate at group size = 10 was the highest and was significantly higher than those in other conditions (*p*s < 0.05). Group size = 10 reached the satisfactory cutoff with a mean coverage rate of 0.91. The coverage was the lowest at group size = 200.

### Estimating υ without finite sample adjustment to β_b_

Results suggest that the performance of ${\tilde{\upsilon }}_{OPE}$ was influenced by the size of the *b* path. According to Equation 16, the finite sample adjustment of $\hat{\upsilon }$ has two parts: (1) Adjusting β_*b*_ and (2) adjusting $R_{Y,MX}^{2}$ and $r_{YX}^{2}$. $R_{Y,MX}^{2}$ and $r_{YX}^{2}$ were unlikely to be influenced by the size of *b* path. Therefore, we conducted additional analyses which estimated υ using Equation 14 without finite sample adjustment to β_*b*_ and with the Olkin–Pratt extended shrinkage method for $R_{Y,MX}^{2}$ and $r_{YX}^{2}$. The results are provided in the online supplements. Similar to the previous results of the ${\tilde{\upsilon }}_{OPE}$ with adjusted β_*b*_ (Equation 16), the size of the *b* path had significant effects on the bias, standardized bias, and MSE of this estimator. We conclude that this estimator did not further improve ${\tilde{\upsilon }}_{OPE}$.

## Empirical example

We present an empirical example to help facilitate the interpretation of the υ effect size measure with a multicategorical predictor. The data were acquired through the NIMH-funded (PI: Rivera Mindt; K23MH079718) Medication Adherence Study at the Icahn School of Medicine at Mount Sinai (ISMMS) in New York City. We were interested in the mediation process between race/ethnicity and antiretroviral medication adherence *via* perceived racial and ethnic discrimination. There were 90 participants with completed measures. Race/ethnicity was a multicategorical variable with four groups: non-Hispanic white (group 1; *n*_1_ = 26), Hispanic (group 2; *n*_2_ = 37), Afro-Hispanic (group 3; *n*_3_ = 12), and others (group 4: *n*_4_ = 15). Dummy coding was used and non-Hispanic white was the reference group. The mediator, perceived discrimination (*M*_1_ = 5.04, *SD*_1_ = 1.34; *M*_2_ = 5.27, *SD*_2_ = 0.93; *M*_3_ = 5.25, *SD*_3_ = 0.97; *M*_4_ = 5.40, *SD*_4_ = 0.91), was measured by the Perceived Ethnic Discrimination Questionnaire—Community Version Brief (PEDQ-CVB, scaled from 1 to 5; Brondolo et al., 2005). The outcome variable, medication adherence (*M*_1_ = 77.44, *SD*_1_ = 19.26; *M*_2_ = 61.88, *SD*_2_ = 35.57; *M*_3_ = 61.90, *SD*_3_ = 32.44; *M*_4_ = 52.35, *SD*_4_ = 32.57), was calculated as the percentage (0 to 100%) of doses taken on schedule as assessed by the Medication Event Monitoring System (MEMS; Group AARDEX, 2022).

Table 4 presents the results for unadjusted $\hat{\upsilon }$s and finite sample adjusted $\tilde{\upsilon }$s by correcting ${\hat{\beta }}_{b}$ and different *R*^2^ shrinkage methods. Similar to simulation results, all *R*^2^ shrinkage methods were performed equally, with $\tilde{\upsilon }$ ranging from 0.146 to 0.147, meaning the variance of medication adherence explained by race/ethnicity *via* perceived discrimination was equal to 0.15. All adjusted $\tilde{\upsilon }$s had similar 95% bootstrapped confidence intervals [−0.23, 1.55].

**Table 4:** Estimates of υ for mediation model in empirical example.

**Effect**	**Unstandardized estimate**	**95% CI [LL, UL]**	***t***	***p***
â_1_ ([Hispanic - non-Hispanic White] → Perceived Mediation)	0.152	[−0.348, 0.651]	0.60	0.55
â_2_ ([Afro-Hispanic - non-Hispanic White] → Perceived Mediation)	0.141	[−0.560, 0.842]	0.40	0.69
â_3_ ([Others - non-Hispanic White] → Perceived Mediation)	0.146	[−0.507, 0.799]	0.44	0.66
$\hat{b}$ (Perceived Mediation → Medication Adherence)	3.30	[−2.864, 9.465]	1.06	0.29
ĉ_1_ ([Hispanic - non-Hispanic White] → Medication Adherence)	−16.328	[−32.019, −0.637]	−2.07	0.042
ĉ_2_ ([Afro-Hispanic - non-Hispanic White] → Medication Adherence)	−16.239	[−37.588, 5.110]	−1.51	0.13
ĉ_3_ ([Others - non-Hispanic White] → Medication Adherence)	−26.283	[−46.206, −6.360]	−2.62	0.010
Unadjusted $\hat{\upsilon }$	263.661	[1.880, 1394.482]		
${\tilde{\upsilon }}_{Claudy}$	0.146	[−0.227, 1.550]		
${\tilde{\upsilon }}_{Ezekiel}$	0.147	[−0.227, 1.551]		
${\tilde{\upsilon }}_{OP}$	0.146	[−0.227, 1.549]		
${\tilde{\upsilon }}_{OPE}$	0.146	[−0.228, 1.549]		
${\tilde{\upsilon }}_{Pratt}$	0.146	[−0.228, 1.549]		
${\tilde{\upsilon }}_{Smith}$	0.146	[−0.227, 1.551]		
${\tilde{\upsilon }}_{Walker}$	0.146	[−0.227, 1.550]		
${\tilde{\upsilon }}_{Wherry}$	0.147	[−0.226, 1.552]		

## Discussion

The goal of this study was to extend the mediation effect size υ developed by Lachowicz et al. (2018) to mediation models involving a multicategorical predictor. Theoretically, υ has many desirable properties as a mediation effect size, particularly for its monotonicity, and bootstrapping can be used to construct its confidence interval. Their simulation showed that the unadjusted and finite sample adjusted sample estimators were consistent effect size measures. Furthermore, this effect size measure is standardized with an invariance of a linear transformation, so it is independent of the predictor scales, the mediator, and the outcome variable. We applied υ to a mediation model with a multicategorical predictor. In this scenario, our simulation results showed that υ did not retain some of the desiderata asserted by Lachowicz et al. (2018). Based on our results, the size of *b* path was the most important factor that negatively influenced the accuracy of the sample estimator of υ. In our data generation process, there were no large differences between the effect sizes of the *a* path and those of the *b* path. On further scrutiny, the performance of the sample estimator with uncorrected ${\hat{\beta }}_{b}$ was similar to the sample estimator with the ${\hat{\beta }}_{b}$ correction. Therefore, the large effect of *b* path remained undiscovered. *R*^2^ shrinkage methods produced slightly less biased υ estimates. The *R*^2^ shrinkage methods performed similarly on adjusting ${\tilde{R}}_{Y,MX}^{2}$ and ${\tilde{r}}_{YX}^{2}$. *R*^2^ shrinkage methods might not be the source of high values of bias, standardized bias, and MSE.

Since ${\tilde{\upsilon }}_{OPE}$ achieved higher performance among all the sample estimators of υ across all the experimental conditions, it is recommended that researchers use ${\tilde{\upsilon }}_{OPE}$ for simple mediation models involving a multicategorical predictor, as illustrated earlier. However, researchers should be cautious about the scenarios mentioned below:

(1) When the size of *b* path reaches 0.39–0.59, ${\tilde{\upsilon }}_{OPE}$ could become an upwardly biased effect size measure. A large *b* path (e.g., *b* = 0.59) could also result in a large value of MSE in ${\tilde{\upsilon }}_{OPE}$.
(2) ${\tilde{\upsilon }}_{OPE}$ does not perform well and will be positively biased when *a*_*i*_ paths have small effects (i.e., Cohen's *d* close or equal to 0).
(3) Researchers should pay attention to small group sizes. ${\tilde{\upsilon }}_{OPE}$ could have high standardized bias and high MSE when *n* = 10.

There are several limitations to this study. First, further analyses should be conducted on the relationship between the effect of the size of *b* path and the bias of the sample estimator of υ. Second, since this effect size measure is appropriate for the mediation model specified in this study, future studies should further develop this effect size measure to be suitable for more complex mediation models, such as models with multicategorical mediators or outcomes, models with moderations or latent variables, and models with multilevel or longitudinal data structures. Finally, since there are more rigorous assumptions that need to be made to justify an indirect effect as a causal effect, it is possible for researchers to introduce unknown bias into the estimation of υ. Future studies should investigate the performance of the estimator when such moderate violations of assumptions occur.

## Data availability statement

The raw data supporting the conclusions of this article will be made available by the authors, without undue reservation.

## Ethics statement

The studies involving human participants were reviewed and approved by NIMH-funded study (Grant# K23MH079718; Principal Investigator: M. Rivera Mindt) at the Icahn School of Medicine at Mount Sinai (ISMMS) https://reporter.nih.gov/search/XK3XEZYfbEuZQESg5r-YNA/project-details/7681039. The patients/participants provided their written informed consent to participate in this study.

## Author contributions

All authors listed have made a substantial, direct, and intellectual contribution to the work and approved it for publication.
