# Supplementary material for: Effect size measure for mediation analysis with a multicategorical predictor
Source: Front Psychol. 2023 Mar 10;14:1101440. doi: 10.3389/fpsyg.2023.1101440 (PMC10036747; doi:10.3389/fpsyg.2023.1101440)
Supplement: Supplementary file 1 [file Data_Sheet_1.docx]

**Online Supplementary Materials**

**Table S1**

*ANOVA of Bias of* $\tilde{\upsilon}_{OPE}$

|  | *df* | *MS* | *F* | *p* | $\eta^{2}$ |
| --- | --- | --- | --- | --- | --- |
| 1. Number of groups | 2 | .00535 | 3.20 | .04 | .009 |
| Residuals | 717 | .00167 |  |  |  |
|  |  |  |  |  |  |
| 2. Sample size per group | 4 | .00096 | .57 | .69 | .003 |
| Residuals | 715 | .00168 |  |  |  |
|  |  |  |  |  |  |
| 3. Effect size of *a*_i_ paths | 3 | .05673 | 39.13 | < .0001 | .141 |
| Residuals | 716 | .00145 |  |  |  |
|  |  |  |  |  |  |
| 4. Size of *b* path | 3 | .26281 | 448.22 | < .0001 | .653 |
| Residuals | 716 | .00059 |  |  |  |
|  |  |  |  |  |  |
| 5. Effect size of $c_{i}^{'}$ paths | 2 | .01360 | 8.26 | .0003 | .023 |
| Residuals | 717 | .00165 |  |  |  |

**Table S2**

*Multiple Comparisons of Bias of* $\tilde{\upsilon}_{OPE}$

| Comparison | Mean Difference | *p* adjusted |
| --- | --- | --- |
| Number of groups |  |  |
| 4 - 3 | -.005 | .350 |
| 5 - 3 | -.009 | .030 |
| 5 - 4 | -.004 | .480 |
|  |  |  |
| Effect size of *a*_i_ paths |  |  |
| .2 – 0 | .000 | 1.000 |
| .5 – 0 | -.014 | .004 |
| .8 – 0 | -.038 | < .0001 |
| .5 - .2 | -.013 | .006 |
| .8 - .2 | -.037 | < .0001 |
| .8 - .5 | -.024 | < .0001 |
|  |  |  |
| Size of *b* path |  |  |
| .15 - 0 | -.003 | .530 |
| .39 - 0 | .007 | .049 |
| .59 - 0 | .077 | < .0001 |
| .39 - .15 | .010 | .001 |
| .59 - .15 | .080 | < .0001 |
| .59 - .39 | .070 | < .0001 |
|  |  |  |
| Effect size of $c_{i}^{'}$ paths | |  |
| .2 - .1 | .007 | .150 |
| .3 - .1 | .015 | .0002 |
| .3 - .2 | .008 | .070 |

*Note*. Tukey’s HSD adjustment was used.

**Table S3**

*ANOVA of Standardized Bias of* $\tilde{\upsilon}_{OPE}$

|  | *df* | *MS* | *F* | *p* | $\eta^{2}$ |
| --- | --- | --- | --- | --- | --- |
| 1. Number of groups | 2 | 48.97535 | 2.84 | .059 | .008 |
| Residuals | 717 | 17.26553 |  |  |  |
|  |  |  |  |  |  |
| 2. Sample size per group | 4 | 128.13216 | 7.66 | < .0001 | .041 |
| Residuals | 715 | 16.73400 |  |  |  |
|  |  |  |  |  |  |
| 3. Effect size of *a*_i_ paths | 3 | 321.33668 | 19.98 | < .0001 | .077 |
| Residuals | 716 | 16.08006 |  |  |  |
|  |  |  |  |  |  |
| 4. Size of *b* path | 3 | 404.67726 | 25.73 | < .0001 | .097 |
| Residuals | 716 | 15.73087 |  |  |  |
|  |  |  |  |  |  |
| 5. Effect size of $c_{i}^{'}$ paths | 2 | 4.37241 | .25 | .778 | .001 |
| Residuals | 717 | 17.38994 |  |  |  |

**Table S4**

*Multiple Comparisons of Standardized Bias of* $\tilde{\upsilon}_{OPE}$

| Comparison | Mean Difference | *p* adjusted |
| --- | --- | --- |
| Sample size per group |  |  |
| 100 – 10 | -2.1714 | < .0001 |
| 200 – 10 | -2.1542 | < .0001 |
| 25 – 10 | -1.8523 | .001 |
| 50 – 10 | -2.1713 | < .0001 |
| 200 – 100 | .0172 | 1.000 |
| 25 – 100 | .3191 | .964 |
| 50 – 100 | .0001 | 1.000 |
| 25 – 200 | .3019 | .971 |
| 50 – 200 | -.0171 | 1.000 |
| 50 – 25 | -.3190 | .964 |
| Effect size of *a*_i_ paths |  |  |
| .2 – 0 | -.0897 | .997 |
| .5 – 0 | -1.3198 | .010 |
| .8 – 0 | -2.8554 | < .0001 |
| .5 - .2 | -1.2300 | .019 |
| .8 - .2 | -2.7656 | < .0001 |
| .8 - .5 | -1.5356 | .002 |
| Size of *b* path |  |  |
| .15 – 0 | -3.3491 | < .0001 |
| .39 – 0 | -1.6814 | .0003 |
| .59 – 0 | -.4460 | .710 |
| .39 – .15 | 1.6677 | .0004 |
| .59 – .15 | 2.9031 | < .0001 |
| .59 – .39 | 1.2355 | .017 |

*Note*. Tukey’s HSD adjustment was used.

**Table S5**

*ANOVA of MSE of* $\tilde{\upsilon}_{OPE}$

|  | *df* | *MS* | *F* | *p* | $\eta^{2}$ |
| --- | --- | --- | --- | --- | --- |
| 1. Number of groups | 2 | .00007 | 1.11 | .33 | .003 |
| Residuals | 717 | .00007 |  |  |  |
|  |  |  |  |  |  |
| 2. Sample size per group | 4 | .00135 | 23.18 | < .0001 | .115 |
| Residuals | 715 | .00006 |  |  |  |
|  |  |  |  |  |  |
| 3. Effect size of *a*_i_ paths | 3 | .00006 | .90 | .44 | .004 |
| Residuals | 716 | .00007 |  |  |  |
|  |  |  |  |  |  |
| 4. Size of *b* path | 3 | .00874 | 301.85 | < .0001 | .558 |
| Residuals | 716 | .00003 |  |  |  |
|  |  |  |  |  |  |
| 5. Effect size of $c_{i}^{'}$ paths | 2 | .00015 | 2.29 | .10 | .006 |
| Residuals | 717 | .00007 |  |  |  |

**Table S6**

*Multiple Comparisons of MSE of* $\tilde{\upsilon}_{OPE}$

| Comparison | Mean Difference | *p* adjusted |
| --- | --- | --- |
| Sample size per group |  |  |
| 100 – 10 | -.007 | < .0001 |
| 200 – 10 | -.007 | < .0001 |
| 25 – 10 | -.005 | < .0001 |
| 50 – 10 | -.007 | < .0001 |
| 200 – 100 | .000 | 1.000 |
| 25 – 100 | .002 | .330 |
| 50 – 100 | .000 | .980 |
| 25 – 200 | .002 | .200 |
| 50 – 200 | .001 | .930 |
| 50 – 25 | -.001 | .670 |
|  |  |  |
| Size of *b* path |  |  |
| .15 – 0 | .000 | 1.000 |
| .39 – 0 | .002 | .0008 |
| .59 – 0 | .015 | < .0001 |
| .39 – .15 | .002 | .002 |
| .59 – .15 | .014 | < .0001 |
| .59 – .39 | .012 | < .0001 |

*Note*. Tukey’s HSD adjustment was used.

**Table S7**

*ANOVA of Coverage Rate of* $\tilde{\upsilon}_{OPE}$

|  | *df* | *MS* | *F* | *p* | $\eta^{2}$ |
| --- | --- | --- | --- | --- | --- |
| 1. Number of groups | 2 | .25425 | 1.83 | .161 | .005 |
| Residuals | 717 | .13904 |  |  |  |
|  |  |  |  |  |  |
| 2. Sample size per group | 4 | 4.22423 | 36.26 | < .0001 | .169 |
| Residuals | 715 | .11650 |  |  |  |
|  |  |  |  |  |  |
| 3. Effect size of *a*_i_ paths | 3 | 2.61152 | 2.24 | < .0001 | .078 |
| Residuals | 716 | .12900 |  |  |  |
|  |  |  |  |  |  |
| 4. Size of *b* path | 3 | 9.80510 | 99.18 | < .0001 | .294 |
| Residuals | 716 | .09886 |  |  |  |
|  |  |  |  |  |  |
| 5. Effect size of $c_{i}^{'}$ paths | 2 | .00105 | .01 | .993 | < .0001 |
| Residuals | 717 | .13974 |  |  |  |

**Table S8**

*Multiple Comparisons of Coverage Rate of* $\tilde{\upsilon}_{OPE}$

| Comparison | Mean Difference | *p* adjusted |
| --- | --- | --- |
| Sample size per group |  |  |
| 100 – 10 | -.3386 | < .0001 |
| 200 – 10 | -.4407 | < .0001 |
| 25 – 10 | -.1394 | .005 |
| 50 – 10 | -.2345 | < .0001 |
| 200 – 100 | -.1021 | .083 |
| 25 – 100 | .1993 | < .0001 |
| 50 – 100 | .1041 | .074 |
| 25 – 200 | .3014 | < .0001 |
| 50 – 200 | .2062 | < .0001 |
| 50 – 25 | -.0951 | .126 |
| Effect size of *a*_i_ paths |  |  |
| .2 – 0 | .1554 | < .0001 |
| .5 – 0 | .2819 | < .0001 |
| .8 – 0 | .2155 | < .0001 |
| .5 - .2 | .1265 | .005 |
| .8 - .2 | .0601 | .387 |
| .8 - .5 | -.0665 | .296 |
| Size of *b* path |  |  |
| .15 – 0 | -.3118 | < .0001 |
| .39 – 0 | -.4274 | < .0001 |
| .59 – 0 | -.5423 | < .0001 |
| .39 – .15 | -.1156 | .003 |
| .59 – .15 | -.2305 | < .0001 |
| .59 – .39 | -.1149 | .003 |

*Note*. Tukey’s HSD adjustment was used.

**Table S9**

*Population Value of* $\upsilon$ *in Simulation Conditions*

|  |  |  | $\upsilon$ | | |
| --- | --- | --- | --- | --- | --- |
| Effect Size of *a*_i_ Paths | Size of *b* Path | Effect Size of $c_{i}^{'}$ Paths | *k* = 3 | *k* = 4 | *k* = 5 |
| 0 | 0 | .1 | .0000 | .0000 | .0000 |
|  |  | .2 | .0000 | .0000 | .0000 |
|  |  | .3 | .0000 | .0000 | .0000 |
|  | .15 | .1 | .0000 | .0000 | .0000 |
|  |  | .2 | .0000 | .0000 | .0000 |
|  |  | .3 | .0000 | .0000 | .0000 |
|  | .39 | .1 | .0000 | .0000 | .0000 |
|  |  | .2 | .0000 | .0000 | .0000 |
|  |  | .3 | .0000 | .0000 | .0000 |
|  | .59 | .1 | .0000 | .0000 | .0000 |
|  |  | .2 | .0000 | .0000 | .0000 |
|  |  | .3 | .0000 | .0000 | .0000 |
| .2 | 0 | .1 | .0000 | .0000 | .0000 |
|  |  | .2 | .0000 | .0000 | .0000 |
|  |  | .3 | .0000 | .0000 | .0000 |
|  | .15 | .1 | .0006 | .0011 | .0017 |
|  |  | .2 | .0006 | .00100 | .0016 |
|  |  | .3 | .0006 | .00100 | .0014 |
|  | .39 | .1 | .0034 | .0064 | .0100 |
|  |  | .2 | .0033 | .0061 | .0092 |
|  |  | .3 | .0032 | .0057 | .0085 |
|  | .59 | .1 | .0067 | .0122 | .0193 |
|  |  | .2 | .0066 | .0119 | .0179 |
|  |  | .3 | .0063 | .0112 | .0164 |
| .5 | 0 | .1 | .0000 | .0000 | .0000 |
|  |  | .2 | .0000 | .0000 | .0000 |
|  |  | .3 | .0000 | .0000 | .0000 |
|  | .15 | .1 | .0036 | .0067 | .0104 |
|  |  | .2 | .0035 | .0063 | .0096 |
|  |  | .3 | .0034 | .0058 | .0087 |
|  | .39 | .1 | .021 | .0377 | .0572 |
|  |  | .2 | .0203 | .0355 | .0522 |
|  |  | .3 | .0191 | .0326 | .0464 |
|  | .59 | .1 | .0400 | .0705 | .1053 |
|  |  | .3 | .0366 | .0609 | .0847 |
| .8 | 0 | .1 | .0000 | .0000 | .0000 |
|  |  | .2 | .0000 | .0000 | .0000 |
|  |  | .3 | .0000 | .0000 | .0000 |
|  | .15 | .1 | .0092 | .0168 | .0260 |
|  |  | .2 | .0088 | .0159 | .0236 |
|  |  | .3 | .0085 | .0144 | .0210 |
|  | .39 | .1 | .0516 | .0892 | .1301 |
|  |  | .2 | .0487 | .0822 | .1163 |
|  |  | .3 | .0461 | .0752 | .1022 |
|  | .59 | .1 | .0948 | .1578 | .2217 |
|  |  | .2 | .0893 | .1456 | .1982 |
|  |  | .3 | .0852 | .1329 | .1752 |

**Table S10**

*ANOVAs of Bias of All Other Sample Estimators of* $\upsilon$ *Besides* $\tilde{\upsilon}_{OPE}$

| Sample Estimator | Source | *df* | *MS* | *F* | *p* | $\eta^{2}$ |
| --- | --- | --- | --- | --- | --- | --- |
| $\hat{\upsilon}$ | 1. Number of groups | 2 | .00494 | 2.75 | .060 | .008 |
|  | Residuals | 717 | .00180 |  |  |  |
|  |  |  |  |  |  |  |
|  | 2. Sample size per group | 4 | .00371 | 2.06 | .080 | .010 |
|  | Residuals | 715 | .00180 |  |  |  |
|  |  |  |  |  |  |  |
|  | 3. Effect size of *a*_i_ paths | 3 | .05856 | 37.35 | < .0001 | .135 |
|  | Residuals | 716 | .00157 |  |  |  |
|  |  |  |  |  |  |  |
|  | 4. Size of *b* path | 3 | .28492 | 459.85 | < .0001 | .658 |
|  | Residuals | 716 | .00062 |  |  |  |
|  |  |  |  |  |  |  |
|  | 5. Effect size of $c_{i}^{'}$ paths | 2 | .01429 | 8.07 | .0003 | .022 |
|  | Residuals | 717 | .00177 |  |  |  |
|  |  |  |  |  |  |  |
| $\tilde{\upsilon}_{Claudy}$ | 1. Number of groups | 2 | .00633 | 3.60 | .028 | .010 |
|  | Residuals | 717 | .00176 |  |  |  |
|  |  |  |  |  |  |  |
|  | 2. Sample size per group | 4 | .00228 | 1.29 | .272 | .007 |
|  | Residuals | 715 | .00177 |  |  |  |
|  |  |  |  |  |  |  |
|  | 3. Effect size of *a*_i_ paths | 3 | .05764 | 37.50 | < .0001 | .136 |
|  | Residuals | 716 | .00154 |  |  |  |
|  |  |  |  |  |  |  |
|  | 4. Size of *b* path | 3 | .27875 | 456.58 | < .0001 | .657 |
|  | Residuals | 716 | .00061 |  |  |  |
|  |  |  |  |  |  |  |
|  | 5. Effect size of $c_{i}^{'}$ paths | 2 | .01331 | 7.65 | .001 | .021 |
|  | Residuals | 717 | .00174 |  |  |  |
|  |  |  |  |  |  |  |
| $\tilde{\upsilon}_{\mathrm{Ezekiel}}$ | 1. Number of groups | 2 | .00637 | 3.64 | .027 | .010 |
|  | Residuals | 717 | .00175 |  |  |  |
|  |  |  |  |  |  |  |
|  | 2. Sample size per group | 4 | .00205 | 1.16 | .326 | .006 |
|  | Residuals | 715 | .00176 |  |  |  |
|  |  |  |  |  |  |  |
|  | 3. Effect size of *a*_i_ paths | 3 | .05890 | 38.64 | < .0001 | .139 |
|  | Residuals | 716 | .00152 |  |  |  |
|  |  |  |  |  |  |  |
|  | 4. Size of *b* path | 3 | .27574 | 447.91 | < .0001 | .652 |
|  | Residuals | 716 | .00062 |  |  |  |
|  |  |  |  |  |  |  |
|  | 5. Effect size of $c_{i}^{'}$ paths | 2 | .01291 | 7.45 | .001 | .020 |
|  | Residuals | 717 | .00173 |  |  |  |
|  |  |  |  |  |  |  |
| $\tilde{\upsilon}_{OP}$ | 1. Number of groups | 2 | .00564 | 3.34 | .036 | .009 |
|  | Residuals | 717 | .00169 |  |  |  |
|  |  |  |  |  |  |  |
|  | 2. Sample size per group | 4 | .00127 | .75 | .560 | .004 |
|  | Residuals | 715 | .00170 |  |  |  |
|  |  |  |  |  |  |  |
|  | 3. Effect size of *a*_i_ paths | 3 | .05706 | 38.88 | < .0001 | .140 |
|  | Residuals | 716 | .00147 |  |  |  |
|  |  |  |  |  |  |  |
|  | 4. Size of *b* path | 3 | .26582 | 448.26 | < .0001 | .653 |
|  | Residuals | 716 | .00059 |  |  |  |
|  |  |  |  |  |  |  |
|  | 5. Effect size of $c_{i}^{'}$ paths | 2 | .01349 | 8.09 | .0003 | .022 |
|  | Residuals | 717 | .00167 |  |  |  |
|  |  |  |  |  |  |  |
| $\tilde{\upsilon}_{\mathrm{Pratt}}$ | 1. Number of groups | 2 | .00547 | 3.27 | .039 | .009 |
|  | Residuals | 717 | .00167 |  |  |  |
|  |  |  |  |  |  |  |
|  | 2. Sample size per group | 4 | .00107 | .63 | .640 | .004 |
|  | Residuals | 715 | .00169 |  |  |  |
|  |  |  |  |  |  |  |
|  | 3. Effect size of *a*_i_ paths | 3 | .05689 | 39.14 | < .0001 | .141 |
|  | Residuals | 716 | .00145 |  |  |  |
|  |  |  |  |  |  |  |
|  | 4. Size of *b* path | 3 | .26331 | 447.33 | < .0001 | .652 |
|  | Residuals | 716 | .00059 |  |  |  |
|  |  |  |  |  |  |  |
|  | 5. Effect size of $c_{i}^{'}$ paths | 2 | .01354 | 8.20 | .0003 | .022 |
|  | Residuals | 717 | .00165 |  |  |  |
|  |  |  |  |  |  |  |
| $\tilde{\upsilon}_{\mathrm{Smith}}$ | 1. Number of groups | 2 | .00616 | 3.51 | .030 | .010 |
|  | Residuals | 717 | .00175 |  |  |  |
|  |  |  |  |  |  |  |
|  | 2. Sample size per group | 4 | .00179 | 1.02 | .398 | .006 |
|  | Residuals | 715 | .00177 |  |  |  |
|  |  |  |  |  |  |  |
|  | 3. Effect size of *a*_i_ paths | 3 | .05887 | 38.56 | < .0001 | .139 |
|  | Residuals | 716 | .00153 |  |  |  |
|  |  |  |  |  |  |  |
|  | 4. Size of *b* path | 3 | .27678 | 45.87 | < .0001 | .654 |
|  | Residuals | 716 | .00061 |  |  |  |
|  |  |  |  |  |  |  |
|  | 5. Effect size of $c_{i}^{'}$ paths | 2 | .01293 | 7.45 | .001 | .020 |
|  | Residuals | 717 | .00174 |  |  |  |
|  |  |  |  |  |  |  |
| $\tilde{\upsilon}_{\mathrm{Walker}}$ | 1. Number of groups | 2 | .00643 | 3.64 | .027 | .010 |
|  | Residuals | 717 | .00177 |  |  |  |
|  |  |  |  |  |  |  |
|  | 2. Sample size per group | 4 | .00246 | 1.38 | .238 | .008 |
|  | Residuals | 715 | .00178 |  |  |  |
|  |  |  |  |  |  |  |
|  | 3. Effect size of *a*_i_ paths | 3 | .05773 | 37.30 | < .0001 | .135 |
|  | Residuals | 716 | .00155 |  |  |  |
|  |  |  |  |  |  |  |
|  | 4. Size of *b* path | 3 | .28072 | 457.59 | < .0001 | .657 |
|  | Residuals | 716 | .00061 |  |  |  |
|  |  |  |  |  |  |  |
|  | 5. Effect size of $c_{i}^{'}$ paths | 2 | .01328 | 7.59 | .001 | .021 |
|  | Residuals | 717 | .00175 |  |  |  |
|  |  |  |  |  |  |  |
| $\tilde{\upsilon}_{\mathrm{Wherry}}$ | 1. Number of groups | 2 | .00678 | 3.72 | .025 | .010 |
|  | Residuals | 717 | .00182 |  |  |  |
|  |  |  |  |  |  |  |
|  | 2. Sample size per group | 4 | .00283 | 1.55 | .187 | .009 |
|  | Residuals | 715 | .00183 |  |  |  |
|  |  |  |  |  |  |  |
|  | 3. Effect size of *a*_i_ paths | 3 | .05940 | 37.27 | < .0001 | .135 |
|  | Residuals | 716 | .00159 |  |  |  |
|  |  |  |  |  |  |  |
|  | 4. Size of *b* path | 3 | .28903 | 457.56 | < .0001 | .657 |
|  | Residuals | 716 | .00063 |  |  |  |
|  |  |  |  |  |  |  |
|  | 5. Effect size of $c_{i}^{'}$ paths | 2 | .01277 | 7.08 | .001 | .019 |
|  | Residuals | 717 | .00180 |  |  |  |

**Table S11**

*Multiple Comparisons of Bias for All Other Sample Estimators of* $\upsilon$ *Besides* $\tilde{\upsilon}_{OPE}$

| Sample Estimator | Comparison | Mean Difference | *p* adjusted |
| --- | --- | --- | --- |
| $\hat{\upsilon}$ | Effect size of *a*_i_ paths |  |  |
|  | .2 – 0 | -.001 | .998 |
|  | .5 – 0 | -.014 | .004 |
|  | .8 – 0 | -.039 | < .0001 |
|  | .5 - .2 | -.014 | .007 |
|  | .8 - .2 | -.038 | < .0001 |
|  | .8 - .5 | -.024 | < .0001 |
|  | Size of *b* path |  |  |
|  | .15 - 0 | -.003 | .740 |
|  | .39 - 0 | .010 | .001 |
|  | .59 - 0 | .081 | < .0001 |
|  | .39 - .15 | .012 | < .0001 |
|  | .59 - .15 | .084 | < .0001 |
|  | .59 - .39 | .071 | < .0001 |
|  | Effect size of $c_{i}^{'}$ paths | |  |
|  | .2 - .1 | .007 | .148 |
|  | .3 - .1 | .015 | < .0001 |
|  | .3 - .2 | .008 | .082 |
| $\tilde{\upsilon}_{Claudy}$ | Number of groups |  |  |
|  | 4 - 3 | -.006 | .301 |
|  | 5 - 3 | -.010 | .021 |
|  | 5 - 4 | -.005 | .455 |
|  | Effect size of *a*_i_ paths |  |  |
|  | .2 – 0 | -.001 | .999 |
|  | .5 – 0 | -.014 | .005 |
|  | .8 – 0 | -.038 | < .0001 |
|  | .5 - .2 | -.013 | .007 |
|  | .8 - .2 | -.038 | < .0001 |
|  | .8 - .5 | -.024 | < .0001 |
|  | Size of *b* path |  |  |
|  | .15 - 0 | -.003 | .613 |
|  | .39 - 0 | .008 | .011 |
|  | .59 - 0 | .080 | < .0001 |
|  | .39 - .15 | .011 | < .0001 |
|  | .59 - .15 | .083 | < .0001 |
|  | .59 - .39 | .072 | < .0001 |
|  | Effect size of $c_{i}^{'}$ paths | |  |
|  | .2 - .1 | .007 | .171 |
|  | .3 - .1 | .015 | < .0001 |
|  | .3 - .2 | .008 | .088 |
| $\tilde{\upsilon}_{\mathrm{Ezekiel}}$ | Number of groups |  |  |
|  | 4 - 3 | -.006 | .300 |
|  | 5 - 3 | -.010 | .020 |
|  | 5 - 4 | -.005 | .448 |
|  | Effect size of *a*_i_ paths |  |  |
|  | .2 – 0 | -.001 | .999 |
|  | .5 – 0 | -.014 | .004 |
|  | .8 – 0 | -.039 | < .0001 |
|  | .5 - .2 | -.014 | .006 |
|  | .8 - .2 | -.038 | < .0001 |
|  | .8 - .5 | -.025 | < .0001 |
|  | Size of *b* path |  |  |
|  | .15 - 0 | -.003 | .632 |
|  | .39 - 0 | .008 | .010 |
|  | .59 - 0 | .079 | < .0001 |
|  | .39 - .15 | .011 | < .0001 |
|  | .59 - .15 | .082 | < .0001 |
|  | .59 - .39 | .071 | < .0001 |
|  | Effect size of $c_{i}^{'}$ paths | |  |
|  | .2 - .1 | .007 | .180 |
|  | .3 - .1 | .015 | < .0001 |
|  | .3 - .2 | .008 | .094 |
| $\tilde{\upsilon}_{OP}$ | Number of groups |  |  |
|  | 4 - 3 | -.005 | .333 |
|  | 5 - 3 | -.010 | .027 |
|  | 5 - 4 | -.004 | .474 |
|  | Effect size of *a*_i_ paths |  |  |
|  | .2 – 0 | .000 | .999 |
|  | .5 – 0 | -.014 | .004 |
|  | .8 – 0 | -.038 | < .0001 |
|  | .5 - .2 | -.013 | .006 |
|  | .8 - .2 | -.038 | < .0001 |
|  | .8 - .5 | -.024 | < .0001 |
|  | Size of *b* path |  |  |
|  | .15 - 0 | -.003 | .550 |
|  | .39 - 0 | .007 | .036 |
|  | .59 - 0 | .078 | < .0001 |
|  | .39 - .15 | .010 | < .0001 |
|  | .59 - .15 | .081 | < .0001 |
|  | .59 - .39 | .071 | < .0001 |
|  | Effect size of $c_{i}^{'}$ paths | |  |
|  | .2 - .1 | .007 | .155 |
|  | .3 - .1 | .015 | < .0001 |
|  | .3 - .2 | .008 | .077 |
| $\tilde{\upsilon}_{\mathrm{Pratt}}$ | Number of groups |  |  |
|  | 4 - 3 | -.005 | .344 |
|  | 5 - 3 | -.010 | .029 |
|  | 5 - 4 | -.004 | .480 |
|  | Effect size of *a*_i_ paths |  |  |
|  | .2 – 0 | .000 | .999 |
|  | .5 – 0 | -.014 | .004 |
|  | .8 – 0 | -.038 | < .0001 |
|  | .5 - .2 | -.013 | .006 |
|  | .8 - .2 | -.037 | < .0001 |
|  | .8 - .5 | -.024 | < .0001 |
|  | Size of *b* path |  |  |
|  | .15 - 0 | -.003 | .535 |
|  | .39 - 0 | .007 | .046 |
|  | .59 - 0 | .077 | < .0001 |
|  | .39 - .15 | .010 | < .0001 |
|  | .59 - .15 | .081 | < .0001 |
|  | .59 - .39 | .070 | < .0001 |
|  | Effect size of $c_{i}^{'}$ paths | |  |
|  | .2 - .1 | .007 | .151 |
|  | .3 - .1 | .015 | < .0001 |
|  | .3 - .2 | .008 | .075 |
| $\tilde{\upsilon}_{\mathrm{Smith}}$ | Number of groups |  |  |
|  | 4 - 3 | -.006 | .316 |
|  | 5 - 3 | -.010 | .023 |
|  | 5 - 4 | -.005 | .456 |
|  | Effect size of *a*_i_ paths |  |  |
|  | .2 – 0 | -.001 | .999 |
|  | .5 – 0 | -.014 | .004 |
|  | .8 – 0 | -.039 | < .0001 |
|  | .5 - .2 | -.014 | .006 |
|  | .8 - .2 | -.038 | < .0001 |
|  | .8 - .5 | -.025 | < .0001 |
|  | Size of *b* path |  |  |
|  | .15 - 0 | -.003 | .635 |
|  | .39 - 0 | .008 | .009 |
|  | .59 - 0 | .080 | < .0001 |
|  | .39 - .15 | .011 | < .0001 |
|  | .59 - .15 | .083 | < .0001 |
|  | .59 - .39 | .071 | < .0001 |
|  | Effect size of $c_{i}^{'}$ paths | |  |
|  | .2 - .1 | .007 | .180 |
|  | .3 - .1 | .015 | < .0001 |
|  | .3 - .2 | .008 | .094 |
| $\tilde{\upsilon}_{\mathrm{Walker}}$ | Number of groups |  |  |
|  | 4 - 3 | -.006 | .297 |
|  | 5 - 3 | -.010 | .020 |
|  | 5 - 4 | -.005 | .452 |
|  | Effect size of *a*_i_ paths |  |  |
|  | .2 – 0 | -.001 | .999 |
|  | .5 – 0 | -.014 | .005 |
|  | .8 – 0 | -.038 | < .0001 |
|  | .5 - .2 | -.013 | .007 |
|  | .8 - .2 | -.038 | < .0001 |
|  | .8 - .5 | -.024 | < .0001 |
|  | Size of *b* path |  |  |
|  | .15 - 0 | -.003 | .622 |
|  | .39 - 0 | .008 | .009 |
|  | .59 - 0 | .080 | < .0001 |
|  | .39 - .15 | .011 | < .0001 |
|  | .59 - .15 | .083 | < .0001 |
|  | .59 - .39 | .072 | < .0001 |
|  | Effect size of $c_{i}^{'}$ paths | |  |
|  | .2 - .1 | .007 | .174 |
|  | .3 - .1 | .015 | < .0001 |
|  | .3 - .2 | .008 | .090 |
| $\tilde{\upsilon}_{\mathrm{Wherry}}$ | Number of groups |  |  |
|  | 4 - 3 | -.006 | .291 |
|  | 5 - 3 | -.011 | .018 |
|  | 5 - 4 | -.005 | .440 |
|  | Effect size of *a*_i_ paths |  |  |
|  | .2 – 0 | -.001 | .999 |
|  | .5 – 0 | -.014 | .004 |
|  | .8 – 0 | -.039 | < .0001 |
|  | .5 - .2 | -.014 | .007 |
|  | .8 - .2 | -.038 | < .0001 |
|  | .8 - .5 | -.025 | < .0001 |
|  | Size of *b* path |  |  |
|  | .15 - 0 | -.003 | .690 |
|  | .39 - 0 | .009 | .003 |
|  | .59 - 0 | .082 | < .0001 |
|  | .39 - .15 | .012 | < .0001 |
|  | .59 - .15 | .085 | < .0001 |
|  | .59 - .39 | .072 | < .0001 |
|  | Effect size of $c_{i}^{'}$ paths | |  |
|  | .2 - .1 | .007 | .196 |
|  | .3 - .1 | .015 | .001 |
|  | .3 - .2 | .008 | .105 |

*Note*. Tukey’s HSD adjustment was used.

**Table S12**

*ANOVAs of Standardized Bias of All Other Sample Estimators of* $\upsilon$ *Besides* $\tilde{\upsilon}_{OPE}$

| Sample Estimator | Source | *df* | *MS* | *F* | *p* | $\eta^{2}$ |
| --- | --- | --- | --- | --- | --- | --- |
| $\hat{\upsilon}$ | 1. Number of groups | 2 | 21.01889 | 5.39 | .0048 | .015 |
|  | Residuals | 717 | 3.89988 |  |  |  |
|  |  |  |  |  |  |  |
|  | 2. Sample size per group | 4 | 17.51050 | 4.52 | .0013 | .025 |
|  | Residuals | 715 | 3.87163 |  |  |  |
|  |  |  |  |  |  |  |
|  | 3. Effect size of *a*_i_ paths | 3 | 188.19381 | 59.26 | < .0001 | .199 |
|  | Residuals | 716 | 3.17552 |  |  |  |
|  |  |  |  |  |  |  |
|  | 4. Size of *b* path | 3 | 177.22195 | 55.01 | < .0001 | .187 |
|  | Residuals | 716 | 3.22149 |  |  |  |
|  |  |  |  |  |  |  |
|  | 5. Effect size of $c_{i}^{'}$ paths | 2 | 55.73612 | 14.66 | < .0001 | .039 |
|  | Residuals | 717 | 3.80304 |  |  |  |
|  |  |  |  |  |  |  |
| $\tilde{\upsilon}_{Claudy}$ | 1. Number of groups | 2 | 43.14269 | 2.89 | .0564 | .008 |
|  | Residuals | 717 | 14.94070 |  |  |  |
|  |  |  |  |  |  |  |
|  | 2. Sample size per group | 4 | 116.69197 | 8.08 | < .0001 | .043 |
|  | Residuals | 715 | 14.45035 |  |  |  |
|  |  |  |  |  |  |  |
|  | 3. Effect size of *a*_i_ paths | 3 | 316.45505 | 23.00 | < .0001 | .088 |
|  | Residuals | 716 | 13.75615 |  |  |  |
|  |  |  |  |  |  |  |
|  | 4. Size of *b* path | 3 | 360.17248 | 26.54 | < .0001 | .100 |
|  | Residuals | 716 | 13.57298 |  |  |  |
|  |  |  |  |  |  |  |
|  | 5. Effect size of $c_{i}^{'}$ paths | 2 | 5.27291 | .35 | .7045 | .001 |
|  | Residuals | 717 | 15.04634 |  |  |  |
|  |  |  |  |  |  |  |
| $\tilde{\upsilon}_{\mathrm{Ezekiel}}$ | 1. Number of groups | 2 | 36.56026 | 2.70 | .0679 | .007 |
|  | Residuals | 717 | 13.54051 |  |  |  |
|  |  |  |  |  |  |  |
|  | 2. Sample size per group | 4 | 104.55926 | 7.98 | < .0001 | .043 |
|  | Residuals | 715 | 13.09571 |  |  |  |
|  |  |  |  |  |  |  |
|  | 3. Effect size of *a*_i_ paths | 3 | 316.84033 | 25.69 | < .0001 | .097 |
|  | Residuals | 716 | 12.33400 |  |  |  |
|  |  |  |  |  |  |  |
|  | 4. Size of *b* path | 3 | 338.30697 | 27.63 | < .0001 | .104 |
|  | Residuals | 716 | 12.24406 |  |  |  |
|  |  |  |  |  |  |  |
|  | 5. Effect size of $c_{i}^{'}$ paths | 2 | 6.36345 | .47 | .6270 | .001 |
|  | Residuals | 717 | 13.62474 |  |  |  |
|  |  |  |  |  |  |  |
| $\tilde{\upsilon}_{OP}$ | 1. Number of groups | 2 | 48.66360 | 2.86 | .0581 | .008 |
|  | Residuals | 717 | 17.02890 |  |  |  |
|  |  |  |  |  |  |  |
|  | 2. Sample size per group | 4 | 128.45864 | 7.79 | < .0001 | .042 |
|  | Residuals | 715 | 16.49401 |  |  |  |
|  |  |  |  |  |  |  |
|  | 3. Effect size of *a*_i_ paths | 3 | 321.63103 | 20.30 | < .0001 | .078 |
|  | Residuals | 716 | 15.84100 |  |  |  |
|  |  |  |  |  |  |  |
|  | 4. Size of *b* path | 3 | 398.34706 | 25.67 | < .0001 | .097 |
|  | Residuals | 716 | 15.51956 |  |  |  |
|  |  |  |  |  |  |  |
|  | 5. Effect size of $c_{i}^{'}$ paths | 2 | 4.39209 | .26 | .7742 | .001 |
|  | Residuals | 717 | 17.15239 |  |  |  |
|  |  |  |  |  |  |  |
| $\tilde{\upsilon}_{\mathrm{Pratt}}$ | 1. Number of groups | 2 | 51.83569 | 2.91 | .0549 | .008 |
|  | Residuals | 717 | 17.78539 |  |  |  |
|  |  |  |  |  |  |  |
|  | 2. Sample size per group | 4 | 136.17555 | 7.91 | < .0001 | .042 |
|  | Residuals | 715 | 17.21831 |  |  |  |
|  |  |  |  |  |  |  |
|  | 3. Effect size of *a*_i_ paths | 3 | 322.06191 | 19.39 | < .0001 | .075 |
|  | Residuals | 716 | 16.60560 |  |  |  |
|  |  |  |  |  |  |  |
|  | 4. Size of *b* path | 3 | 409.90580 | 25.24 | < .0001 | .096 |
|  | Residuals | 716 | 16.23754 |  |  |  |
|  |  |  |  |  |  |  |
|  | 5. Effect size of $c_{i}^{'}$ paths | 2 | 4.05966 | .23 | .7973 | .001 |
|  | Residuals | 717 | 17.91866 |  |  |  |
|  |  |  |  |  |  |  |
| $\tilde{\upsilon}_{\mathrm{Smith}}$ | 1. Number of groups | 2 | 18.64245 | 1.74 | .1768 | .005 |
|  | Residuals | 717 | 10.73472 |  |  |  |
|  |  |  |  |  |  |  |
|  | 2. Sample size per group | 4 | 66.01903 | 6.32 | .0001 | .034 |
|  | Residuals | 715 | 10.44756 |  |  |  |
|  |  |  |  |  |  |  |
|  | 3. Effect size of *a*_i_ paths | 3 | 314.15655 | 33.12 | < .0001 | .122 |
|  | Residuals | 716 | 9.48549 |  |  |  |
|  |  |  |  |  |  |  |
|  | 4. Size of *b* path | 3 | 301.89675 | 31.66 | < .0001 | .117 |
|  | Residuals | 716 | 9.53686 |  |  |  |
|  |  |  |  |  |  |  |
|  | 5. Effect size of $c_{i}^{'}$ paths | 2 | 12.70608 | 1.18 | .3073 | .003 |
|  | Residuals | 717 | 10.75128 |  |  |  |
|  |  |  |  |  |  |  |
| $\tilde{\upsilon}_{\mathrm{Walker}}$ | 1. Number of groups | 2 | 42.42834 | 2.89 | .0560 | .008 |
|  | Residuals | 717 | 14.65915 |  |  |  |
|  |  |  |  |  |  |  |
|  | 2. Sample size per group | 4 | 115.03883 | 8.12 | < .0001 | .043 |
|  | Residuals | 715 | 14.17526 |  |  |  |
|  |  |  |  |  |  |  |
|  | 3. Effect size of *a*_i_ paths | 3 | 315.68583 | 23.43 | < .0001 | .089 |
|  | Residuals | 716 | 13.47544 |  |  |  |
|  |  |  |  |  |  |  |
|  | 4. Size of *b* path | 3 | 354.93781 | 26.67 | < .0001 | .100 |
|  | Residuals | 716 | 13.31097 |  |  |  |
|  |  |  |  |  |  |  |
|  | 5. Effect size of $c_{i}^{'}$ paths | 2 | 5.44499 | .37 | .6917 | .001 |
|  | Residuals | 717 | 14.76231 |  |  |  |
|  |  |  |  |  |  |  |
| $\tilde{\upsilon}_{\mathrm{Wherry}}$ | 1. Number of groups | 2 | 17.46905 | 1.78 | .1695 | .005 |
|  | Residuals | 717 | 9.81824 |  |  |  |
|  |  |  |  |  |  |  |
|  | 2. Sample size per group | 4 | 61.76240 | 6.47 | < .0001 | .035 |
|  | Residuals | 715 | 9.54905 |  |  |  |
|  |  |  |  |  |  |  |
|  | 3. Effect size of *a*_i_ paths | 3 | 309.66271 | 36.08 | < .0001 | .131 |
|  | Residuals | 716 | 8.58328 |  |  |  |
|  |  |  |  |  |  |  |
|  | 4. Size of *b* path | 3 | 282.12539 | 32.43 | < .0001 | .120 |
|  | Residuals | 716 | 8.69866 |  |  |  |
|  |  |  |  |  |  |  |
|  | 5. Effect size of $c_{i}^{'}$ paths | 2 | 14.25482 | 1.45 | .2351 | .004 |
|  | Residuals | 717 | 9.82721 |  |  |  |

**Table S13**

*Multiple Comparisons of Standardized Bias for All Other Sample Estimators of* $\upsilon$ *Besides* $\tilde{\upsilon}_{OPE}$

| Sample Estimator | Comparison | Mean Difference | *p* adjusted |
| --- | --- | --- | --- |
| $\hat{\upsilon}$ | Number of groups |  |  |
|  | 4 - 3 | .222 | .436 |
|  | 5 - 3 | .586 | .003 |
|  | 5 - 4 | .364 | .108 |
|  | Sample size per group |  |  |
|  | 100 – 10 | -.811 | .005 |
|  | 200 – 10 | -.834 | .003 |
|  | 25 – 10 | -.633 | .051 |
|  | 50 – 10 | -.762 | .009 |
|  | 200 – 100 | -.023 | 1.000 |
|  | 25 – 100 | .178 | .940 |
|  | 50 – 100 | .049 | 1.000 |
|  | 25 – 200 | .201 | .909 |
|  | 50 – 200 | .073 | .998 |
|  | 50 – 25 | -.129 | .981 |
|  | Effect size of *a*_i_ paths |  |  |
|  | .2 – 0 | -.091 | .962 |
|  | .5 – 0 | -.903 | < .0001 |
|  | .8 – 0 | -2.208 | < .0001 |
|  | .5 - .2 | -.812 | < .0001 |
|  | .8 - .2 | -2.117 | < .0001 |
|  | .8 - .5 | -1.306 | < .0001 |
|  | Size of *b* path |  |  |
|  | .15 - 0 | -1.530 | < .0001 |
|  | .39 - 0 | -.516 | .033 |
|  | .59 - 0 | .842 | .0001 |
|  | .39 - .15 | 1.014 | < .0001 |
|  | .59 - .15 | 2.372 | < .0001 |
|  | .59 - .39 | 1.358 | < .0001 |
|  | Effect size of $c_{i}^{'}$ paths | |  |
|  | .2 - .1 | .479 | .020 |
|  | .3 - .1 | .964 | < .0001 |
|  | .3 - .2 | .485 | .018 |
| $\tilde{\upsilon}_{Claudy}$ | Sample size per group |  |  |
|  | 100 – 10 | -2.075 | < .0001 |
|  | 200 – 10 | -2.061 | < .0001 |
|  | 25 – 10 | -1.750 | .001 |
|  | 50 – 10 | -2.070 | < .0001 |
|  | 200 – 100 | .014 | 1.000 |
|  | 25 – 100 | .326 | .950 |
|  | 50 – 100 | .005 | 1.000 |
|  | 25 – 200 | .311 | .958 |
|  | 50 – 200 | -.009 | 1.000 |
|  | 50 – 25 | -.321 | .953 |
|  | Effect size of *a*_i_ paths |  |  |
|  | .2 – 0 | -.112 | .992 |
|  | .5 – 0 | -1.318 | .004 |
|  | .8 – 0 | -2.845 | < .0001 |
|  | .5 - .2 | -1.207 | .011 |
|  | .8 - .2 | -2.733 | < .0001 |
|  | .8 - .5 | -1.526 | .001 |
|  | Size of *b* path |  |  |
|  | .15 - 0 | -3.089 | < .0001 |
|  | .39 - 0 | -1.458 | .001 |
|  | .59 - 0 | -.236 | .930 |
|  | .39 - .15 | 1.6308 | .0002 |
|  | .59 - .15 | 2.853 | < .0001 |
|  | .59 - .39 | 1.222 | .009 |
| $\tilde{\upsilon}_{\mathrm{Ezekiel}}$ | Sample size per group |  |  |
|  | 100 – 10 | -1.965 | < .0001 |
|  | 200 – 10 | -1.9505 | .0001 |
|  | 25 – 10 | -1.648 | .001 |
|  | 50 – 10 | -1.962 | < .0001 |
|  | 200 – 100 | .014 | 1.000 |
|  | 25 – 100 | .317 | .946 |
|  | 50 – 100 | .003 | 1.000 |
|  | 25 – 200 | .302 | .954 |
|  | 50 – 200 | -.012 | 1.000 |
|  | 50 – 25 | -.314 | .948 |
|  | Effect size of *a*_i_ paths |  |  |
|  | .2 – 0 | -.129 | .985 |
|  | .5 – 0 | -1.326 | .002 |
|  | .8 – 0 | -2.855 | < .0001 |
|  | .5 - .2 | -1.197 | .007 |
|  | .8 - .2 | -2.726 | < .0001 |
|  | .8 - .5 | -1.5289 | .0002 |
|  | Size of *b* path |  |  |
|  | .15 - 0 | -2.951 | < .0001 |
|  | .39 - 0 | -1.345 | .002 |
|  | .59 - 0 | -.128 | .986 |
|  | .39 - .15 | 1.6059 | .0001 |
|  | .59 - .15 | 2.822 | < .0001 |
|  | .59 - .39 | 1.216 | .006 |
| $\tilde{\upsilon}_{OP}$ | Sample size per group |  |  |
|  | 100 – 10 | -2.1746 | .0001 |
|  | 200 – 10 | -2.1576 | .0001 |
|  | 25 – 10 | -1.852 | .001 |
|  | 50 – 10 | -2.1737 | .0001 |
|  | 200 – 100 | .017 | 1.000 |
|  | 25 – 100 | .322 | .962 |
|  | 50 – 100 | .001 | 1.000 |
|  | 25 – 200 | .305 | .969 |
|  | 50 – 200 | -.016 | 1.000 |
|  | 50 – 25 | -.321 | .963 |
|  | Effect size of *a*_i_ paths |  |  |
|  | .2 – 0 | -.093 | .996 |
|  | .5 – 0 | -1.322 | .009 |
|  | .8 – 0 | -2.858 | < .0001 |
|  | .5 - .2 | -1.229 | .018 |
|  | .8 - .2 | -2.765 | < .0001 |
|  | .8 - .5 | -1.536 | .002 |
|  | Size of *b* path |  |  |
|  | .15 - 0 | -3.315 | < .0001 |
|  | .39 - 0 | -1.6520 | .0004 |
|  | .59 - 0 | -.419 | .744 |
|  | .39 - .15 | 1.663 | < .0001 |
|  | .59 - .15 | 2.895 | < .0001 |
|  | .59 - .39 | 1.233 | .016 |
| $\tilde{\upsilon}_{\mathrm{Pratt}}$ | Sample size per group |  |  |
|  | 100 – 10 | -2.2381 | .0001 |
|  | 200 – 10 | -2.2211 | .0001 |
|  | 25 – 10 | -1.914 | .001 |
|  | 50 – 10 | -2.2369 | .0001 |
|  | 200 – 100 | .017 | 1.000 |
|  | 25 – 100 | .324 | .964 |
|  | 50 – 100 | .001 | 1.000 |
|  | 25 – 200 | .307 | .970 |
|  | 50 – 200 | -.016 | 1.000 |
|  | 50 – 25 | -.323 | .965 |
|  | Effect size of *a*_i_ paths |  |  |
|  | .2 – 0 | -.088 | .997 |
|  | .5 – 0 | -1.321 | .012 |
|  | .8 – 0 | -2.858 | < .0001 |
|  | .5 - .2 | -1.233 | .022 |
|  | .8 - .2 | -2.770 | < .0001 |
|  | .8 - .5 | -1.537 | .002 |
|  | Size of *b* path |  |  |
|  | .15 - 0 | -3.385 | < .0001 |
|  | .39 - 0 | -1.7236 | .0003 |
|  | .59 - 0 | -.490 | .657 |
|  | .39 - .15 | 1.661 | .001 |
|  | .59 - .15 | 2.895 | < .0001 |
|  | .59 - .39 | 1.234 | .020 |
| $\tilde{\upsilon}_{\mathrm{Smith}}$ | Sample size per group |  |  |
|  | 100 – 10 | -1.5580 | .0005 |
|  | 200 – 10 | -1.5376 | .0006 |
|  | 25 – 10 | -1.2999 | .006 |
|  | 50 – 10 | -1.5766 | .0004 |
|  | 200 – 100 | .0204 | 1.000 |
|  | 25 – 100 | .2581 | .961 |
|  | 50 – 100 | -.0186 | 1.000 |
|  | 25 – 200 | .2377 | .971 |
|  | 50 – 200 | -.0390 | 1.000 |
|  | 50 – 25 | -.2767 | .950 |
|  | Effect size of *a*_i_ paths |  |  |
|  | .2 – 0 | -.1448 | .970 |
|  | .5 – 0 | -1.3278 | .0003 |
|  | .8 – 0 | -2.8502 | < .0001 |
|  | .5 - .2 | -1.1830 | .002 |
|  | .8 - .2 | -2.7054 | < .0001 |
|  | .8 - .5 | -1.5224 | < .0001 |
|  | Size of *b* path |  |  |
|  | .15 - 0 | -2.6066 | < .0001 |
|  | .39 - 0 | -.9688 | .016 |
|  | .59 - 0 | .2503 | .869 |
|  | .39 - .15 | 1.6378 | < .0001 |
|  | .59 - .15 | 2.8569 | < .0001 |
|  | .59 - .39 | 1.2191 | .001 |
| $\tilde{\upsilon}_{\mathrm{Walker}}$ | Sample size per group |  |  |
|  | 100 – 10 | -2.061 | < .0001 |
|  | 200 – 10 | -2.047 | < .0001 |
|  | 25 – 10 | -1.735 | .001 |
|  | 50 – 10 | -2.055 | < .0001 |
|  | 200 – 100 | .014 | 1.000 |
|  | 25 – 100 | .326 | .948 |
|  | 50 – 100 | .005 | 1.000 |
|  | 25 – 200 | .312 | .956 |
|  | 50 – 200 | -.008 | 1.000 |
|  | 50 – 25 | -.321 | .951 |
|  | Effect size of *a*_i_ paths |  |  |
|  | .2 – 0 | -.114 | .991 |
|  | .5 – 0 | -1.318 | .004 |
|  | .8 – 0 | -2.842 | < .0001 |
|  | .5 - .2 | -1.203 | .010 |
|  | .8 - .2 | -2.728 | < .0001 |
|  | .8 - .5 | -1.525 | .001 |
|  | Size of *b* path |  |  |
|  | .15 - 0 | -3.056 | < .0001 |
|  | .39 - 0 | -1.430 | .001 |
|  | .59 - 0 | -.209 | .948 |
|  | .39 - .15 | 1.6262 | .0002 |
|  | .59 - .15 | 2.847 | < .0001 |
|  | .59 - .39 | 1.220 | .009 |
| $\tilde{\upsilon}_{\mathrm{Wherry}}$ | Sample size per group |  |  |
|  | 100 – 10 | -1.5094 | .0004 |
|  | 200 – 10 | -1.4921 | .0004 |
|  | 25 – 10 | -1.244 | .006 |
|  | 50 – 10 | -1.5218 | .0003 |
|  | 200 – 100 | .017 | 1.000 |
|  | 25 – 100 | .266 | .950 |
|  | 50 – 100 | -.012 | 1.000 |
|  | 25 – 200 | .249 | .960 |
|  | 50 – 200 | -.030 | 1.000 |
|  | 50 – 25 | -.278 | .941 |
|  | Effect size of *a*_i_ paths |  |  |
|  | .2 – 0 | -.156 | .958 |
|  | .5 – 0 | -1.3219 | .0001 |
|  | .8 – 0 | -2.836 | < .0001 |
|  | .5 - .2 | -1.166 | .001 |
|  | .8 - .2 | -2.680 | < .0001 |
|  | .8 - .5 | -1.514 | < .0001 |
|  | Size of *b* path |  |  |
|  | .15 - 0 | -2.444 | < .0001 |
|  | .39 - 0 | -.835 | .037 |
|  | .59 - 0 | .375 | .623 |
|  | .39 - .15 | 1.609 | < .0001 |
|  | .59 - .15 | 2.819 | < .0001 |
|  | .59 - .39 | 1.210 | .001 |

*Note*. Tukey’s HSD adjustment was used.

**Table S14**

*ANOVAs of MSE of All Other Sample Estimators of* $\upsilon$ *Besides* $\tilde{\upsilon}_{OPE}$

| Sample Estimator | Source | *df* | *MS* | *F* | *p* | $\eta^{2}$ |
| --- | --- | --- | --- | --- | --- | --- |
| $\hat{\upsilon}$ | 1. Number of groups | 2 | .00009 | 1.55 | .213 | .004 |
|  | Residuals | 717 | .00006 |  |  |  |
|  |  |  |  |  |  |  |
|  | 2. Sample size per group | 4 | .00137 | 25.72 | < .0001 | .126 |
|  | Residuals | 715 | .00005 |  |  |  |
|  |  |  |  |  |  |  |
|  | 3. Effect size of *a*_i_ paths | 3 | .00020 | 3.30 | .020 | .014 |
|  | Residuals | 716 | .00006 |  |  |  |
|  |  |  |  |  |  |  |
|  | 4. Size of *b* path | 3 | .00812 | 301.87 | < .0001 | .558 |
|  | Residuals | 716 | .00003 |  |  |  |
|  |  |  |  |  |  |  |
|  | 5. Effect size of $c_{i}^{'}$ paths | 2 | .00012 | 2.01 | .135 | .006 |
|  | Residuals | 717 | .00006 |  |  |  |
|  |  |  |  |  |  |  |
| $\tilde{\upsilon}_{Claudy}$ | 1. Number of groups | 2 | .00011 | 1.46 | .233 | .004 |
|  | Residuals | 717 | .00008 |  |  |  |
|  |  |  |  |  |  |  |
|  | 2. Sample size per group | 4 | .00168 | 25.32 | < .0001 | .124 |
|  | Residuals | 715 | .00007 |  |  |  |
|  |  |  |  |  |  |  |
|  | 3. Effect size of *a*_i_ paths | 3 | .00008 | 1.00 | .392 | .004 |
|  | Residuals | 716 | .00008 |  |  |  |
|  |  |  |  |  |  |  |
|  | 4. Size of *b* path | 3 | .00966 | 272.89 | < .0001 | .533 |
|  | Residuals | 716 | .00004 |  |  |  |
|  |  |  |  |  |  |  |
|  | 5. Effect size of $c_{i}^{'}$ paths | 2 | .00016 | 2.07 | .127 | .006 |
|  | Residuals | 717 | .00008 |  |  |  |
|  |  |  |  |  |  |  |
| $\tilde{\upsilon}_{\mathrm{Ezekiel}}$ | 1. Number of groups | 2 | .00010 | 1.38 | .252 | .004 |
|  | Residuals | 717 | .00007 |  |  |  |
|  |  |  |  |  |  |  |
|  | 2. Sample size per group | 4 | .00156 | 24.58 | < .0001 | .121 |
|  | Residuals | 715 | .00006 |  |  |  |
|  |  |  |  |  |  |  |
|  | 3. Effect size of *a*_i_ paths | 3 | .00008 | 1.15 | .329 | .005 |
|  | Residuals | 716 | .00007 |  |  |  |
|  |  |  |  |  |  |  |
|  | 4. Size of *b* path | 3 | .00939 | 285.37 | < .0001 | .545 |
|  | Residuals | 716 | .00003 |  |  |  |
|  |  |  |  |  |  |  |
|  | 5. Effect size of $c_{i}^{'}$ paths | 2 | .00015 | 2.03 | .132 | .006 |
|  | Residuals | 717 | .00007 |  |  |  |
|  |  |  |  |  |  |  |
| $\tilde{\upsilon}_{OP}$ | 1. Number of groups | 2 | .00008 | 1.21 | .300 | .003 |
|  | Residuals | 717 | .00007 |  |  |  |
|  |  |  |  |  |  |  |
|  | 2. Sample size per group | 4 | .00142 | 23.78 | < .0001 | .117 |
|  | Residuals | 715 | .00006 |  |  |  |
|  |  |  |  |  |  |  |
|  | 3. Effect size of *a*_i_ paths | 3 | .00006 | .93 | .424 | .004 |
|  | Residuals | 716 | .00007 |  |  |  |
|  |  |  |  |  |  |  |
|  | 4. Size of *b* path | 3 | .00891 | 293.20 | < .0001 | .551 |
|  | Residuals | 716 | .00003 |  |  |  |
|  |  |  |  |  |  |  |
|  | 5. Effect size of $c_{i}^{'}$ paths | 2 | .00015 | 2.22 | .110 | .006 |
|  | Residuals | 717 | .00007 |  |  |  |
|  |  |  |  |  |  |  |
| $\tilde{\upsilon}_{\mathrm{Pratt}}$ | 1. Number of groups | 2 | .00007 | 1.13 | .325 | .003 |
|  | Residuals | 717 | .00007 |  |  |  |
|  |  |  |  |  |  |  |
|  | 2. Sample size per group | 4 | .00136 | 23.28 | < .0001 | .115 |
|  | Residuals | 715 | .00006 |  |  |  |
|  |  |  |  |  |  |  |
|  | 3. Effect size of *a*_i_ paths | 3 | .00006 | .92 | .430 | .004 |
|  | Residuals | 716 | .00007 |  |  |  |
|  |  |  |  |  |  |  |
|  | 4. Size of *b* path | 3 | .00877 | 3.65 | < .0001 | .557 |
|  | Residuals | 716 | .00003 |  |  |  |
|  |  |  |  |  |  |  |
|  | 5. Effect size of $c_{i}^{'}$ paths | 2 | .00015 | 2.27 | .104 | .006 |
|  | Residuals | 717 | .00007 |  |  |  |
|  |  |  |  |  |  |  |
| $\tilde{\upsilon}_{\mathrm{Smith}}$ | 1. Number of groups | 2 | .00010 | 1.37 | .254 | .004 |
|  | Residuals | 717 | .00007 |  |  |  |
|  |  |  |  |  |  |  |
|  | 2. Sample size per group | 4 | .00157 | 24.65 | < .0001 | .121 |
|  | Residuals | 715 | .00006 |  |  |  |
|  |  |  |  |  |  |  |
|  | 3. Effect size of *a*_i_ paths | 3 | .00008 | 1.14 | .333 | .005 |
|  | Residuals | 716 | .00007 |  |  |  |
|  |  |  |  |  |  |  |
|  | 4. Size of *b* path | 3 | .00941 | 284.74 | < .0001 | .544 |
|  | Residuals | 716 | .00003 |  |  |  |
|  |  |  |  |  |  |  |
|  | 5. Effect size of $c_{i}^{'}$ paths | 2 | .00015 | 2.02 | .133 | .006 |
|  | Residuals | 717 | .00007 |  |  |  |
|  |  |  |  |  |  |  |
| $\tilde{\upsilon}_{\mathrm{Walker}}$ | 1. Number of groups | 2 | .00012 | 1.50 | .224 | .004 |
|  | Residuals | 717 | .00008 |  |  |  |
|  |  |  |  |  |  |  |
|  | 2. Sample size per group | 4 | .00173 | 25.55 | < .0001 | .125 |
|  | Residuals | 715 | .00007 |  |  |  |
|  |  |  |  |  |  |  |
|  | 3. Effect size of *a*_i_ paths | 3 | .00008 | 1.01 | .387 | .004 |
|  | Residuals | 716 | .00008 |  |  |  |
|  |  |  |  |  |  |  |
|  | 4. Size of *b* path | 3 | .00978 | 269.98 | < .0001 | .531 |
|  | Residuals | 716 | .00004 |  |  |  |
|  |  |  |  |  |  |  |
|  | 5. Effect size of $c_{i}^{'}$ paths | 2 | .00016 | 2.05 | .130 | .006 |
|  | Residuals | 717 | .00008 |  |  |  |
|  |  |  |  |  |  |  |
| $\tilde{\upsilon}_{\mathrm{Wherry}}$ | 1. Number of groups | 2 | .00013 | 1.60 | .203 | .004 |
|  | Residuals | 717 | .00008 |  |  |  |
|  |  |  |  |  |  |  |
|  | 2. Sample size per group | 4 | .00183 | 26.02 | < .0001 | .127 |
|  | Residuals | 715 | .00007 |  |  |  |
|  |  |  |  |  |  |  |
|  | 3. Effect size of *a*_i_ paths | 3 | .00010 | 1.20 | .309 | .005 |
|  | Residuals | 716 | .00008 |  |  |  |
|  |  |  |  |  |  |  |
|  | 4. Size of *b* path | 3 | .01013 | 267.33 | < .0001 | .528 |
|  | Residuals | 716 | .00004 |  |  |  |
|  |  |  |  |  |  |  |
|  | 5. Effect size of $c_{i}^{'}$ paths | 2 | .00015 | 1.90 | .151 | .005 |
|  | Residuals | 717 | .00008 |  |  |  |

**Table S15**

*Multiple Comparisons of MSE for All Other Sample Estimators of* $\upsilon$ *Besides* $\tilde{\upsilon}_{OPE}$

| Sample Estimator | Comparison | Mean Difference | *p* adjusted |
| --- | --- | --- | --- |
| $\hat{\upsilon}$ | Sample size per group |  |  |
|  | 100 – 10 | -.00720 | < .0001 |
|  | 200 – 10 | -.00744 | < .0001 |
|  | 25 – 10 | -.00536 | < .0001 |
|  | 50 – 10 | -.00665 | < .0001 |
|  | 200 – 100 | -.00024 | .999 |
|  | 25 – 100 | .00183 | .207 |
|  | 50 – 100 | .00055 | .969 |
|  | 25 – 200 | .00208 | .113 |
|  | 50 – 200 | .00079 | .891 |
|  | 50 – 25 | -.00129 | .565 |
|  | Effect size of *a*_i_ paths |  |  |
|  | .2 – 0 | -.00007 | 1.000 |
|  | .5 – 0 | -.00134 | .355 |
|  | .8 – 0 | -.00217 | .041 |
|  | .5 - .2 | -.00127 | .404 |
|  | .8 - .2 | -.00210 | .051 |
|  | .8 - .5 | -.00082 | .744 |
|  | Size of *b* path |  |  |
|  | .15 - 0 | .00018 | .987 |
|  | .39 - 0 | .00256 | < .0001 |
|  | .59 - 0 | .01414 | < .0001 |
|  | .39 - .15 | .00238 | < .0001 |
|  | .59 - .15 | .01396 | < .0001 |
|  | .59 - .39 | .01158 | < .0001 |
| $\tilde{\upsilon}_{Claudy}$ | Sample size per group |  |  |
|  | 100 – 10 | -.00830 | < .0001 |
|  | 200 – 10 | -.00856 | < .0001 |
|  | 25 – 10 | -.00636 | < .0001 |
|  | 50 – 10 | -.00773 | < .0001 |
|  | 200 – 100 | -.00027 | .999 |
|  | 25 – 100 | .00194 | .285 |
|  | 50 – 100 | .00056 | .979 |
|  | 25 – 200 | .00221 | .169 |
|  | 50 – 200 | .00083 | .918 |
|  | 50 – 25 | -.00138 | .632 |
|  | Size of *b* path |  |  |
|  | .15 - 0 | .00013 | .997 |
|  | .39 - 0 | .00239 | .001 |
|  | .59 - 0 | .01568 | < .0001 |
|  | .39 - .15 | .00226 | .003 |
|  | .59 - .15 | .01556 | < .0001 |
|  | .59 - .39 | .01329 | < .0001 |
| $\tilde{\upsilon}_{\mathrm{Ezekiel}}$ | Sample size per group |  |  |
|  | 100 – 10 | -.00767 | < .0001 |
|  | 200 – 10 | -.00793 | < .0001 |
|  | 25 – 10 | -.00586 | < .0001 |
|  | 50 – 10 | -.00714 | < .0001 |
|  | 200 – 100 | -.00025 | .999 |
|  | 25 – 100 | .00182 | .301 |
|  | 50 – 100 | .00053 | .980 |
|  | 25 – 200 | .00207 | .180 |
|  | 50 – 200 | .00078 | .921 |
|  | 50 – 25 | -.00129 | .647 |
|  | Size of *b* path |  |  |
|  | .15 - 0 | .00012 | .997 |
|  | .39 - 0 | .00228 | .001 |
|  | .59 - 0 | .01509 | < .0001 |
|  | .39 - .15 | .00216 | .002 |
|  | .59 - .15 | .01498 | < .0001 |
|  | .59 - .39 | .01281 | < .0001 |
| $\tilde{\upsilon}_{OP}$ | Sample size per group |  |  |
|  | 100 – 10 | -.00732 | < .0001 |
|  | 200 – 10 | -.00756 | < .0001 |
|  | 25 – 10 | -.00561 | < .0001 |
|  | 50 – 10 | -.00682 | < .0001 |
|  | 200 – 100 | -.00024 | .999 |
|  | 25 – 100 | .00171 | .330 |
|  | 50 – 100 | .00050 | .982 |
|  | 25 – 200 | .00195 | .205 |
|  | 50 – 200 | .00073 | .929 |
|  | 50 – 25 | -.00122 | .671 |
|  | Size of *b* path |  |  |
|  | .15 - 0 | .00011 | .997 |
|  | .39 - 0 | .00220 | .001 |
|  | .59 - 0 | .01470 | < .0001 |
|  | .39 - .15 | .00208 | .002 |
|  | .59 - .15 | .01458 | < .0001 |
|  | .59 - .39 | .01250 | < .0001 |
| $\tilde{\upsilon}_{\mathrm{Pratt}}$ | Sample size per group |  |  |
|  | 100 – 10 | -.00715 | < .0001 |
|  | 200 – 10 | -.00739 | < .0001 |
|  | 25 – 10 | -.00546 | < .0001 |
|  | 50 – 10 | -.00666 | < .0001 |
|  | 200 – 100 | -.00024 | .999 |
|  | 25 – 100 | .00170 | .326 |
|  | 50 – 100 | .00050 | .982 |
|  | 25 – 200 | .00193 | .201 |
|  | 50 – 200 | .00073 | .927 |
|  | 50 – 25 | -.00120 | .669 |
|  | Size of *b* path |  |  |
|  | .15 - 0 | .00011 | .997 |
|  | .39 - 0 | .00217 | .001 |
|  | .59 - 0 | .01458 | < .0001 |
|  | .39 - .15 | .00206 | .002 |
|  | .59 - .15 | .01446 | < .0001 |
|  | .59 - .39 | .01240 | < .0001 |
| $\tilde{\upsilon}_{\mathrm{Smith}}$ | Sample size per group |  |  |
|  | 100 – 10 | -.00769 | < .0001 |
|  | 200 – 10 | -.00795 | < .0001 |
|  | 25 – 10 | -.00588 | < .0001 |
|  | 50 – 10 | -.00716 | < .0001 |
|  | 200 – 100 | -.00025 | .999 |
|  | 25 – 100 | .00182 | .301 |
|  | 50 – 100 | .00053 | .980 |
|  | 25 – 200 | .00207 | .181 |
|  | 50 – 200 | .00078 | .921 |
|  | 50 – 25 | -.00129 | .648 |
|  | Size of *b* path |  |  |
|  | .15 - 0 | .00012 | .997 |
|  | .39 - 0 | .00229 | .001 |
|  | .59 - 0 | .01511 | < .0001 |
|  | .39 - .15 | .00216 | .002 |
|  | .59 - .15 | .01499 | < .0001 |
|  | .59 - .39 | .01282 | < .0001 |
| $\tilde{\upsilon}_{\mathrm{Walker}}$ | Sample size per group |  |  |
|  | 100 – 10 | -.00806 | < .0001 |
|  | 200 – 10 | -.00832 | < .0001 |
|  | 25 – 10 | -.00621 | < .0001 |
|  | 50 – 10 | -.00753 | < .0001 |
|  | 200 – 100 | -.00025 | .999 |
|  | 25 – 100 | .00186 | .310 |
|  | 50 – 100 | .00054 | .982 |
|  | 25 – 200 | .00211 | .190 |
|  | 50 – 200 | .00079 | .926 |
|  | 50 – 25 | -.00132 | .653 |
|  | Size of *b* path |  |  |
|  | .15 - 0 | .00012 | .998 |
|  | .39 - 0 | .00232 | .002 |
|  | .59 - 0 | .01540 | < .0001 |
|  | .39 - .15 | .00220 | .003 |
|  | .59 - .15 | .01528 | < .0001 |
|  | .59 - .39 | .01307 | < .0001 |
| $\tilde{\upsilon}_{\mathrm{Wherry}}$ | Sample size per group |  |  |
|  | 100 – 10 | -.00830 | < .0001 |
|  | 200 – 10 | -.00856 | < .0001 |
|  | 25 – 10 | -.00636 | < .0001 |
|  | 50 – 10 | -.00773 | < .0001 |
|  | 200 – 100 | -.00027 | .999 |
|  | 25 – 100 | .00194 | .285 |
|  | 50 – 100 | .00056 | .979 |
|  | 25 – 200 | .00221 | .169 |
|  | 50 – 200 | .00083 | .918 |
|  | 50 – 25 | -.00138 | .632 |
|  | Size of *b* path |  |  |
|  | .15 - 0 | .00013 | .997 |
|  | .39 - 0 | .00239 | .001 |
|  | .59 - 0 | .01568 | < .0001 |
|  | .39 - .15 | .00226 | .003 |
|  | .59 - .15 | .01556 | < .0001 |
|  | .59 - .39 | .01329 | < .0001 |

*Note*. Tukey’s HSD adjustment was used.

**Table S16**

*ANOVAs of Coverage Rate of All Other Sample Estimators of* $\upsilon$ *Besides* $\tilde{\upsilon}_{OPE}$

| Sample Estimator | Source | *df* | *MS* | *F* | *p* | $\eta^{2}$ |
| --- | --- | --- | --- | --- | --- | --- |
| $\hat{\upsilon}$ | 1. Number of groups | 2 | .75567 | 8.48664 | .0002 | .023 |
|  | Residuals | 717 | .08904 |  |  |  |
|  |  |  |  |  |  |  |
|  | 2. Sample size per group | 4 | 3.37041 | 46.4567 | < .0001 | .206 |
|  | Residuals | 715 | .07255 |  |  |  |
|  |  |  |  |  |  |  |
|  | 3. Effect size of *a*_i_ paths | 3 | .5933 | 6.68194 | .0002 | .027 |
|  | Residuals | 716 | .08879 |  |  |  |
|  |  |  |  |  |  |  |
|  | 4. Size of *b* path | 3 | 4.64825 | 64.7378 | < .0001 | .213 |
|  | Residuals | 716 | .0718 |  |  |  |
|  |  |  |  |  |  |  |
|  | 5. Effect size of $c_{i}^{'}$ paths | 2 | .26481 | 2.92895 | .054 | .008 |
|  | Residuals | 717 | .09041 |  |  |  |
|  |  |  |  |  |  |  |
| $\tilde{\upsilon}_{Claudy}$ | 1. Number of groups | 2 | .20933 | 1.48351 | .228 | .004 |
|  | Residuals | 717 | .14111 |  |  |  |
|  |  |  |  |  |  |  |
|  | 2. Sample size per group | 4 | 3.68342 | 3.321 | < .0001 | .145 |
|  | Residuals | 715 | .12148 |  |  |  |
|  |  |  |  |  |  |  |
|  | 3. Effect size of *a*_i_ paths | 3 | 3.33867 | 26.1037 | < .0001 | .099 |
|  | Residuals | 716 | .1279 |  |  |  |
|  |  |  |  |  |  |  |
|  | 4. Size of *b* path | 3 | 1.4607 | 106.677 | < .0001 | .309 |
|  | Residuals | 716 | .09806 |  |  |  |
|  |  |  |  |  |  |  |
|  | 5. Effect size of $c_{i}^{'}$ paths | 2 | .00171 | .01209 | .988 | < .0001 |
|  | Residuals | 717 | .14169 |  |  |  |
|  |  |  |  |  |  |  |
| $\tilde{\upsilon}_{\mathrm{Ezekiel}}$ | 1. Number of groups | 2 | .03494 | .25287 | .777 | .001 |
|  | Residuals | 717 | .13818 |  |  |  |
|  |  |  |  |  |  |  |
|  | 2. Sample size per group | 4 | 2.38174 | 19.0024 | < .0001 | .096 |
|  | Residuals | 715 | .12534 |  |  |  |
|  |  |  |  |  |  |  |
|  | 3. Effect size of *a*_i_ paths | 3 | 4.97304 | 42.276 | < .0001 | .150 |
|  | Residuals | 716 | .11763 |  |  |  |
|  |  |  |  |  |  |  |
|  | 4. Size of *b* path | 3 | 8.82117 | 86.9001 | < .0001 | .267 |
|  | Residuals | 716 | .10151 |  |  |  |
|  |  |  |  |  |  |  |
|  | 5. Effect size of $c_{i}^{'}$ paths | 2 | .1126 | .81617 | .443 | .002 |
|  | Residuals | 717 | .13796 |  |  |  |
|  |  |  |  |  |  |  |
| $\tilde{\upsilon}_{OP}$ | 1. Number of groups | 2 | .24465 | 1.75681 | .173 | .005 |
|  | Residuals | 717 | .13926 |  |  |  |
|  |  |  |  |  |  |  |
|  | 2. Sample size per group | 4 | 4.08253 | 34.7469 | < .0001 | .163 |
|  | Residuals | 715 | .11749 |  |  |  |
|  |  |  |  |  |  |  |
|  | 3. Effect size of *a*_i_ paths | 3 | 2.74749 | 21.3605 | < .0001 | .082 |
|  | Residuals | 716 | .12862 |  |  |  |
|  |  |  |  |  |  |  |
|  | 4. Size of *b* path | 3 | 9.96535 | 101.292 | < .0001 | .298 |
|  | Residuals | 716 | .09838 |  |  |  |
|  |  |  |  |  |  |  |
|  | 5. Effect size of $c_{i}^{'}$ paths | 2 | .00092 | .00658 | .993 | < .0001 |
|  | Residuals | 717 | .13994 |  |  |  |
|  |  |  |  |  |  |  |
| $\tilde{\upsilon}_{\mathrm{Pratt}}$ | 1. Number of groups | 2 | .24976 | 1.79485 | .167 | .005 |
|  | Residuals | 717 | .13915 |  |  |  |
|  |  |  |  |  |  |  |
|  | 2. Sample size per group | 4 | 4.15698 | 35.5339 | < .0001 | .166 |
|  | Residuals | 715 | .11699 |  |  |  |
|  |  |  |  |  |  |  |
|  | 3. Effect size of *a*_i_ paths | 3 | 2.68154 | 2.8177 | < .0001 | .080 |
|  | Residuals | 716 | .12881 |  |  |  |
|  |  |  |  |  |  |  |
|  | 4. Size of *b* path | 3 | 9.87728 | 1.113 | < .0001 | .296 |
|  | Residuals | 716 | .09866 |  |  |  |
|  |  |  |  |  |  |  |
|  | 5. Effect size of $c_{i}^{'}$ paths | 2 | .00101 | .00725 | .993 | < .0001 |
|  | Residuals | 717 | .13985 |  |  |  |
|  |  |  |  |  |  |  |
| $\tilde{\upsilon}_{\mathrm{Smith}}$ | 1. Number of groups | 2 | .26204 | 1.85431 | .157 | .005 |
|  | Residuals | 717 | .14132 |  |  |  |
|  |  |  |  |  |  |  |
|  | 2. Sample size per group | 4 | 3.73694 | 3.7473 | < .0001 | .147 |
|  | Residuals | 715 | .12154 |  |  |  |
|  |  |  |  |  |  |  |
|  | 3. Effect size of *a*_i_ paths | 3 | 3.23569 | 25.1438 | < .0001 | .095 |
|  | Residuals | 716 | .12869 |  |  |  |
|  |  |  |  |  |  |  |
|  | 4. Size of *b* path | 3 | 1.4833 | 106.625 | < .0001 | .309 |
|  | Residuals | 716 | .09832 |  |  |  |
|  |  |  |  |  |  |  |
|  | 5. Effect size of $c_{i}^{'}$ paths | 2 | .00311 | .02189 | .978 | < .0001 |
|  | Residuals | 717 | .14204 |  |  |  |
|  |  |  |  |  |  |  |
| $\tilde{\upsilon}_{\mathrm{Walker}}$ | 1. Number of groups | 2 | .19889 | 1.40633 | .246 | .004 |
|  | Residuals | 717 | .14143 |  |  |  |
|  |  |  |  |  |  |  |
|  | 2. Sample size per group | 4 | 3.59098 | 29.3649 | < .0001 | .141 |
|  | Residuals | 715 | .12229 |  |  |  |
|  |  |  |  |  |  |  |
|  | 3. Effect size of *a*_i_ paths | 3 | 3.476 | 27.2383 | < .0001 | .102 |
|  | Residuals | 716 | .12761 |  |  |  |
|  |  |  |  |  |  |  |
|  | 4. Size of *b* path | 3 | 1.5436 | 107.586 | < .0001 | .311 |
|  | Residuals | 716 | .098 |  |  |  |
|  |  |  |  |  |  |  |
|  | 5. Effect size of $c_{i}^{'}$ paths | 2 | .00236 | .0166 | .984 | < .0001 |
|  | Residuals | 717 | .14197 |  |  |  |
|  |  |  |  |  |  |  |
| $\tilde{\upsilon}_{\mathrm{Wherry}}$ | 1. Number of groups | 2 | .43588 | 3.12159 | .045 | .009 |
|  | Residuals | 717 | .13963 |  |  |  |
|  |  |  |  |  |  |  |
|  | 2. Sample size per group | 4 | 2.75735 | 21.9157 | < .0001 | .109 |
|  | Residuals | 715 | .12582 |  |  |  |
|  |  |  |  |  |  |  |
|  | 3. Effect size of *a*_i_ paths | 3 | 4.2587 | 34.567 | < .0001 | .127 |
|  | Residuals | 716 | .1232 |  |  |  |
|  |  |  |  |  |  |  |
|  | 4. Size of *b* path | 3 | 9.85606 | 98.8088 | < .0001 | .293 |
|  | Residuals | 716 | .09975 |  |  |  |
|  |  |  |  |  |  |  |
|  | 5. Effect size of $c_{i}^{'}$ paths | 2 | .08323 | .5919 | .988 | .002 |
|  | Residuals | 717 | .14062 |  |  |  |

**Table S17**

*Multiple Comparisons of Coverage Rate for All Other Sample Estimators of* $\upsilon$ *Besides* $\tilde{\upsilon}_{OPE}$

| Sample Estimator | Comparison | Mean Difference | *p* adjusted |
| --- | --- | --- | --- |
| $\hat{\upsilon}$ | Number of groups |  |  |
|  | 4 - 3 | -.053 | .127 |
|  | 5 - 3 | -.112 | < .0001 |
|  | 5 - 4 | -.059 | .076 |
|  | Sample size per group |  |  |
|  | 100 – 10 | -.261 | < .0001 |
|  | 200 – 10 | -.382 | < .0001 |
|  | 25 – 10 | -.065 | .252 |
|  | 50 – 10 | -.148 | < .0001 |
|  | 200 – 100 | -.121 | .001 |
|  | 25 – 100 | .196 | < .0001 |
|  | 50 – 100 | .113 | .004 |
|  | 25 – 200 | .317 | < .0001 |
|  | 50 – 200 | .234 | < .0001 |
|  | 50 – 25 | -.083 | .068 |
|  | Effect size of *a*_i_ paths |  |  |
|  | .2 – 0 | .059 | .244 |
|  | .5 – 0 | .140 | < .0001 |
|  | .8 – 0 | .075 | .079 |
|  | .5 - .2 | .081 | .050 |
|  | .8 - .2 | .017 | .952 |
|  | .8 - .5 | -.064 | .172 |
|  | Size of *b* path |  |  |
|  | .15 - 0 | -.119 | < .0001 |
|  | .39 - 0 | -.193 | < .0001 |
|  | .59 - 0 | -.383 | < .0001 |
|  | .39 - .15 | -.074 | .043 |
|  | .59 - .15 | -.264 | < .0001 |
|  | .59 - .39 | -.190 | < .0001 |
| $\tilde{\upsilon}_{Claudy}$ | Sample size per group |  |  |
|  | 100 – 10 | -.311 | < .0001 |
|  | 200 – 10 | -.411 | < .0001 |
|  | 25 – 10 | -.123 | .024 |
|  | 50 – 10 | -.208 | < .0001 |
|  | 200 – 100 | -.101 | .104 |
|  | 25 – 100 | .188 | < .0001 |
|  | 50 – 100 | .103 | .092 |
|  | 25 – 200 | .288 | < .0001 |
|  | 50 – 200 | .203 | < .0001 |
|  | 50 – 25 | -.085 | .235 |
|  | Effect size of *a*_i_ paths |  |  |
|  | .2 – 0 | .180 | < .0001 |
|  | .5 – 0 | .315 | < .0001 |
|  | .8 – 0 | .252 | < .0001 |
|  | .5 - .2 | .135 | .002 |
|  | .8 - .2 | .072 | .225 |
|  | .8 - .5 | -.063 | .336 |
|  | Size of *b* path |  |  |
|  | .15 - 0 | -.312 | < .0001 |
|  | .39 - 0 | -.443 | < .0001 |
|  | .59 - 0 | -.559 | < .0001 |
|  | .39 - .15 | -.130 | .001 |
|  | .59 - .15 | -.247 | < .0001 |
|  | .59 - .39 | -.117 | .002 |
| $\tilde{\upsilon}_{\mathrm{Ezekiel}}$ | Sample size per group |  |  |
|  | 100 – 10 | -.232 | < .0001 |
|  | 200 – 10 | -.326 | < .0001 |
|  | 25 – 10 | -.072 | .413 |
|  | 50 – 10 | -.139 | .008 |
|  | 200 – 100 | -.094 | .165 |
|  | 25 – 100 | .160 | .001 |
|  | 50 – 100 | .093 | .167 |
|  | 25 – 200 | .253 | < .0001 |
|  | 50 – 200 | .187 | < .0001 |
|  | 50 – 25 | -.066 | .506 |
|  | Effect size of *a*_i_ paths |  |  |
|  | .2 – 0 | .240 | < .0001 |
|  | .5 – 0 | .380 | < .0001 |
|  | .8 – 0 | .317 | < .0001 |
|  | .5 - .2 | .140 | .001 |
|  | .8 - .2 | .077 | .145 |
|  | .8 - .5 | -.063 | .303 |
|  | Size of *b* path |  |  |
|  | .15 - 0 | -.280 | < .0001 |
|  | .39 - 0 | -.408 | < .0001 |
|  | .59 - 0 | -.512 | < .0001 |
|  | .39 - .15 | -.128 | .001 |
|  | .59 - .15 | -.232 | < .0001 |
|  | .59 - .39 | -.104 | .011 |
| $\tilde{\upsilon}_{OP}$ | Sample size per group |  |  |
|  | 100 – 10 | -.331 | < .0001 |
|  | 200 – 10 | -.433 | < .0001 |
|  | 25 – 10 | -.134 | .008 |
|  | 50 – 10 | -.227 | < .0001 |
|  | 200 – 100 | -.102 | .086 |
|  | 25 – 100 | .197 | < .0001 |
|  | 50 – 100 | .104 | .077 |
|  | 25 – 200 | .299 | < .0001 |
|  | 50 – 200 | .206 | < .0001 |
|  | 50 – 25 | -.093 | .145 |
|  | Effect size of *a*_i_ paths |  |  |
|  | .2 – 0 | .160 | < .0001 |
|  | .5 – 0 | .289 | < .0001 |
|  | .8 – 0 | .223 | < .0001 |
|  | .5 - .2 | .129 | .004 |
|  | .8 - .2 | .063 | .347 |
|  | .8 - .5 | -.066 | .301 |
|  | Size of *b* path |  |  |
|  | .15 - 0 | -.312 | < .0001 |
|  | .39 - 0 | -.431 | < .0001 |
|  | .59 - 0 | -.547 | < .0001 |
|  | .39 - .15 | -.119 | .002 |
|  | .59 - .15 | -.235 | < .0001 |
|  | .59 - .39 | -.116 | .003 |
| $\tilde{\upsilon}_{\mathrm{Pratt}}$ | Sample size per group |  |  |
|  | 100 – 10 | -.335 | < .0001 |
|  | 200 – 10 | -.437 | < .0001 |
|  | 25 – 10 | -.137 | .006 |
|  | 50 – 10 | -.231 | < .0001 |
|  | 200 – 100 | -.102 | .085 |
|  | 25 – 100 | .198 | < .0001 |
|  | 50 – 100 | .104 | .075 |
|  | 25 – 200 | .300 | < .0001 |
|  | 50 – 200 | .206 | < .0001 |
|  | 50 – 25 | -.094 | .136 |
|  | Effect size of *a*_i_ paths |  |  |
|  | .2 – 0 | .158 | < .0001 |
|  | .5 – 0 | .285 | < .0001 |
|  | .8 – 0 | .219 | < .0001 |
|  | .5 - .2 | .128 | .004 |
|  | .8 - .2 | .061 | .368 |
|  | .8 - .5 | -.066 | .298 |
|  | Size of *b* path |  |  |
|  | .15 - 0 | -.312 | < .0001 |
|  | .39 - 0 | -.429 | < .0001 |
|  | .59 - 0 | -.544 | < .0001 |
|  | .39 - .15 | -.117 | .002 |
|  | .59 - .15 | -.232 | < .0001 |
|  | .59 - .39 | -.115 | .003 |
| $\tilde{\upsilon}_{\mathrm{Smith}}$ | Sample size per group |  |  |
|  | 100 – 10 | -.314 | < .0001 |
|  | 200 – 10 | -.415 | < .0001 |
|  | 25 – 10 | -.126 | .019 |
|  | 50 – 10 | -.211 | < .0001 |
|  | 200 – 100 | -.101 | .101 |
|  | 25 – 100 | .188 | < .0001 |
|  | 50 – 100 | .102 | .093 |
|  | 25 – 200 | .289 | < .0001 |
|  | 50 – 200 | .204 | < .0001 |
|  | 50 – 25 | -.085 | .235 |
|  | Effect size of *a*_i_ paths |  |  |
|  | .2 – 0 | .173 | < .0001 |
|  | .5 – 0 | .310 | < .0001 |
|  | .8 – 0 | .247 | < .0001 |
|  | .5 - .2 | .138 | .002 |
|  | .8 - .2 | .074 | .206 |
|  | .8 - .5 | -.064 | .335 |
|  | Size of *b* path |  |  |
|  | .15 - 0 | -.309 | < .0001 |
|  | .39 - 0 | -.443 | < .0001 |
|  | .59 - 0 | -.559 | < .0001 |
|  | .39 - .15 | -.134 | < .0001 |
|  | .59 - .15 | -.250 | < .0001 |
|  | .59 - .39 | -.116 | .003 |
| $\tilde{\upsilon}_{\mathrm{Walker}}$ | Sample size per group |  |  |
|  | 100 – 10 | -.306 | < .0001 |
|  | 200 – 10 | -.406 | < .0001 |
|  | 25 – 10 | -.119 | .032 |
|  | 50 – 10 | -.203 | < .0001 |
|  | 200 – 100 | -.100 | .108 |
|  | 25 – 100 | .186 | < .0001 |
|  | 50 – 100 | .102 | .095 |
|  | 25 – 200 | .286 | < .0001 |
|  | 50 – 200 | .203 | < .0001 |
|  | 50 – 25 | -.084 | .251 |
|  | Effect size of *a*_i_ paths |  |  |
|  | .2 – 0 | .185 | < .0001 |
|  | .5 – 0 | .321 | < .0001 |
|  | .8 – 0 | .258 | < .0001 |
|  | .5 - .2 | .136 | .002 |
|  | .8 - .2 | .074 | .207 |
|  | .8 - .5 | -.063 | .343 |
|  | Size of *b* path |  |  |
|  | .15 - 0 | -.312 | < .0001 |
|  | .39 - 0 | -.445 | < .0001 |
|  | .59 - 0 | -.561 | < .0001 |
|  | .39 - .15 | -.132 | < .0001 |
|  | .59 - .15 | -.249 | < .0001 |
|  | .59 - .39 | -.116 | .003 |
| $\tilde{\upsilon}_{\mathrm{Wherry}}$ | Number of groups |  |  |
|  | 4 - 3 | -.064 | .143 |
|  | 5 - 3 | -.081 | .048 |
|  | 5 - 4 | -.016 | .884 |
|  | Sample size per group |  |  |
|  | 100 – 10 | -.254 | < .0001 |
|  | 200 – 10 | -.353 | < .0001 |
|  | 25 – 10 | -.086 | .239 |
|  | 50 – 10 | -.158 | .002 |
|  | 200 – 100 | -.099 | .129 |
|  | 25 – 100 | .168 | .001 |
|  | 50 – 100 | .096 | .145 |
|  | 25 – 200 | .267 | < .0001 |
|  | 50 – 200 | .195 | < .0001 |
|  | 50 – 25 | -.072 | .421 |
|  | Effect size of *a*_i_ paths |  |  |
|  | .2 – 0 | .209 | < .0001 |
|  | .5 – 0 | .352 | < .0001 |
|  | .8 – 0 | .292 | < .0001 |
|  | .5 - .2 | .143 | .001 |
|  | .8 - .2 | .083 | .115 |
|  | .8 - .5 | -.060 | .360 |
|  | Size of *b* path |  |  |
|  | .15 - 0 | -.292 | < .0001 |
|  | .39 - 0 | -.431 | < .0001 |
|  | .59 - 0 | -.541 | < .0001 |
|  | .39 - .15 | -.139 | < .0001 |
|  | .59 - .15 | -.249 | < .0001 |
|  | .59 - .39 | -.110 | .005 |

*Note*. Tukey’s HSD adjustment was used.

**Table S18**

*ANOVA of Bias of* $\tilde{\upsilon}_{OPE}$ *with Unadjusted* $\hat{\beta}_{b}$

|  | *df* | *MS* | *F* | *p* | $\eta^{2}$ |
| --- | --- | --- | --- | --- | --- |
| 1. Number of groups | 2 | .00380 | 0.26 | .77 | .001 |
| Residuals | 717 | .01467 |  |  |  |
|  |  |  |  |  |  |
| 2. Sample size per group | 4 | .00016 | 0.01 | 1 | .000 |
| Residuals | 715 | .01472 |  |  |  |
|  |  |  |  |  |  |
| 3. Effect size of *a*_i_ paths | 3 | .05665 | 3.92 | 0.009 | .016 |
| Residuals | 716 | .01447 |  |  |  |
|  |  |  |  |  |  |
| 4. Size of *b* path | 3 | 3.361 | 5413.00 | < .0001 | .958 |
| Residuals | 716 | .001 |  |  |  |
|  |  |  |  |  |  |
| 5. Effect size of $c_{i}^{'}$ paths | 2 | .01366 | .93 | .394 | .003 |
| Residuals | 717 | .01465 |  |  |  |

**Table S19**

*Multiple Comparisons of Bias of* $\tilde{\upsilon}_{OPE}$ *with Unadjusted* $\hat{\beta}_{b}$

| Comparison | Mean Difference | *p* adjusted |
| --- | --- | --- |
| Effect size of *a*_i_ paths |  |  |
| .2 – 0 | -.002 | 1.000 |
| .5 – 0 | -.014 | .067 |
| .8 – 0 | -.038 | .001 |
| .5 - .2 | -.013 | .753 |
| .8 - .2 | -.037 | .002 |
| .8 - .5 | -.024 | .229 |
|  |  |  |
| Size of *b* path |  |  |
| .15 - 0 | .122 | < .0001 |
| .39 - 0 | .240 | < .0001 |
| .59 - 0 | .311 | < .0001 |
| .39 - .15 | .119 | < .0001 |
| .59 - .15 | .189 | < .0001 |
| .59 - .39 | .070 | < .0001 |
|  |  |  |

*Note*. Tukey’s HSD adjustment was used.

**Table S20**

*ANOVA of Standardized Bias of* $\tilde{\upsilon}_{OPE}$ *with Unadjusted* $\hat{\beta}_{b}$

|  | *df* | *MS* | *F* | *p* | $\eta^{2}$ |
| --- | --- | --- | --- | --- | --- |
| 1. Number of groups | 2 | 1693 | 94.25 | < .0001 | .208 |
| Residuals | 717 | 18 |  |  |  |
|  |  |  |  |  |  |
| 2. Sample size per group | 4 | 135.22 | 6.15 | < .0001 | .033 |
| Residuals | 715 | 21.99 |  |  |  |
|  |  |  |  |  |  |
| 3. Effect size of *a*_i_ paths | 3 | 103.54 | 4.65 | .003 | .019 |
| Residuals | 716 | 22.28 |  |  |  |
|  |  |  |  |  |  |
| 4. Size of *b* path | 3 | 2955.1 | 286.10 | < .0001 | .545 |
| Residuals | 716 | 10.3 |  |  |  |
|  |  |  |  |  |  |
| 5. Effect size of $c_{i}^{'}$ paths | 2 | 2.945 | .13 | .878 | .000 |
| Residuals | 717 | 22.672 |  |  |  |

**Table S21**

*Multiple Comparisons of Standardized Bias of* $\tilde{\upsilon}_{OPE}$ *with Unadjusted* $\hat{\beta}_{b}$

| Comparison | Mean Difference | *p* adjusted |
| --- | --- | --- |
| Number of groups |  |  |
| 4 – 3 | 2.047 | < .0001 |
| 5 – 3 | 5.268 | < .0001 |
| 5 – 4 | 3.221 | < .0001 |
|  |  |  |
| Sample size per group |  |  |
| 100 – 10 | 1.7728 | .012 |
| 200 – 10 | 2.4137 | .0001 |
| 25 – 10 | .4865 | .904 |
| 50 – 10 | 1.0580 | .310 |
| 200 – 100 | .6409 | .774 |
| 25 – 100 | 1.2864 | .137 |
| 50 – 100 | .7148 | .695 |
| 25 – 200 | 1.9273 | .005 |
| 50 – 200 | 1.3557 | .102 |
| 50 – 25 | .5716 | .839 |
|  |  |  |
| Effect size of *a*_i_ paths |  |  |
| .2 – 0 | -.147 | 1 |
| .5 – 0 | -.734 | 0.453 |
| .8 – 0 | -1.672 | .005 |
| .5 - .2 | -.587 | 0.64 |
| .8 - .2 | -1.524 | .012 |
| .8 - .5 | -.937 | .236 |
|  |  |  |
| Size of *b* path |  |  |
| .15 - 0 | 2.560 | < .0001 |
| .39 - 0 | 7.118 | < .0001 |
| .59 - 0 | 8.794 | < .0001 |
| .39 - .15 | 4.558 | < .0001 |
| .59 - .15 | 6.234 | < .0001 |
| .59 - .39 | 1.677 | < .0001 |

**Table S22**

*ANOVA of MSE of* $\tilde{\upsilon}_{OPE}$ *with Unadjusted* $\hat{\beta}_{b}$

|  | *df* | *MS* | *F* | *p* | $\eta^{2}$ |
| --- | --- | --- | --- | --- | --- |
| 1. Number of groups | 2 | .0142 | 9.43 | < .0001 | .026 |
| Residuals | 717 | .0015 |  |  |  |
|  |  |  |  |  |  |
| 2. Sample size per group | 4 | .00059 | .38 | .824 | .002 |
| Residuals | 715 | .00155 |  |  |  |
|  |  |  |  |  |  |
| 3. Effect size of *a*_i_ paths | 3 | .01331 | .90 | < .0001 | .036 |
| Residuals | 716 | .00150 |  |  |  |
|  |  |  |  |  |  |
| 4. Size of *b* path | 3 | .3172 | 1427.00 | < .0001 | .857 |
| Residuals | 716 | .0002 |  |  |  |
|  |  |  |  |  |  |
| 5. Effect size of $c_{i}^{'}$ paths | 2 | .00412 | 2.68 | .07 | .007 |
| Residuals | 717 | .00154 |  |  |  |

**Table S23**

*Multiple Comparisons of MSE of* $\tilde{\upsilon}_{OPE}$ *with Unadjusted* $\hat{\beta}_{b}$

| Comparison | Mean Difference | *p* adjusted |
| --- | --- | --- |
| Number of groups |  |  |
| 4 – 3 | -.0112 | .005 |
| 5 – 3 | -.0147 | .0001 |
| 5 – 4 | -.0036 | .56 |
|  |  |  |
| Effect size of *a*_i_ paths |  |  |
| .2 – 0 | -.0003 | 1 |
| .5 – 0 | -.0071 | .300 |
| .8 – 0 | -.0183 | < .0001 |
| .5 - .2 | -.0068 | .335 |
| .8 - .2 | -.0181 | < .0001 |
| .8 - .5 | -.0112 | 0.031 |
|  |  |  |
| Size of *b* path |  |  |
| .15 - 0 | .0109 | < .0001 |
| .39 - 0 | .0519 | < .0001 |
| .59 - 0 | .0920 | < .0001 |
| .39 - .15 | .0410 | < .0001 |
| .59 - .15 | .0810 | < .0001 |
| .59 - .39 | .0401 | < .0001 |

*Note*. Tukey’s HSD adjustment was used.
